# Uev1A promotes breast cancer cell migration by up-regulating *CT45A* expression via the AKT pathway

**DOI:** 10.1186/s12885-021-08750-3

**Published:** 2021-09-09

**Authors:** Tong Niu, Zhaojia Wu, Wei Xiao

**Affiliations:** 1 Beijing Key Laboratory of DNA Damage Responses and College of Life Sciences, Capital Normal University, Beijing, 100048 China; 2 Department of Biochemistry, Microbiology and Immunology, University of Saskatchewan, Saskatoon, SK S7N 5E5 Canada

**Keywords:** Uev1A, CT45A, AKT signaling pathway, NF-κB pathway, Cell migration, EMT

## Abstract

**Background:**

*UEV1A* encodes a ubiquitin-E2 variant closely associated with tumorigenesis and metastasis, but its underlying mechanism in promoting metastasis remains to be investigated.

**Methods:**

In this study, we experimentally manipulated *UEV1A* and *CT45A* gene expression and monitored their effects on cancer-related gene expression, cell migration and the signal transduction cascade.

**Results:**

It was found that *UEV1A* overexpression induces *CT45A* family gene expression in breast cancer cells. Indeed, ectopic expression of *UEV1A* was sufficient to induce *CT45A* and its downstream genes involved in tumorigenesis, epithelial-mesenchymal transition (EMT), stemness and metastasis, and to promote cell migration and EMT signaling. Consistently, depletion of CT45A abolished the above effects, indicating that CT45A is a critical downstream effector of Uev1A. The Uev1A-induced cell migration and EMT signaling was dependent on AKT but independent of NF-κB, indicating that CT45A acts downstream of the AKT pathway.

**Conclusions:**

Based on previous reports and observations in this study, we propose that the Ubc13-Uev1A complex activates AKT through K63-linked polyubiquitination, which leads to enhanced *CT45A* expression, stimulated cell migration and EMT signaling in breast cells. Since similar effects were also observed in a colorectal cancer cell line, the Ubc13/Uev1A-AKT-CT45A axis may also promote tumorigenesis and metastasis in other tissues.

**Supplementary Information:**

The online version contains supplementary material available at 10.1186/s12885-021-08750-3.

## Background

*UEV1*, also known as *CROC1* [1, 2] or *CIR1* [3], was identified as a mammalian homolog of yeast *MMS2* [4], as well as a potential proto-oncogene associated with tumorigenesis and metastasis [5–7]. Indeed, *UEV1* maps to a region (chromosome 20q13.2) where DNA amplification is frequently reported in breast cancers [8–11] and other tumors [12]. Ubiquitin (Ub)-conjugating enzyme variant (UEV, including Mms2 and Uev1 in mammalian cells) is a co-factor of Ubc13 [13] and absolutely required for Ubc13-mediated K63-linked polyubiquitin chain assembly [14–17]. To date, at least three *UEV1* splicing variants have been reported, among which Uev1A and Uev1C could promote K63-linked polyubiquitination by forming a complex with Ubc13, whereas Uev1B could not [18]. Uev1A differs from Uev1C in that it contains 30 additional amino acids at the N-terminus [18, 19].

Despite the fact that Uev1A and Mms2 are two major Uevs in mammalian cells and share a similar biochemical activity, they appear to function differently: Ubc13-Mms2 is required for DNA-damage response, whereas Ubc13-Uev1A is involved in NF-κB activation [18] and AKT activation [6]. Previous studies demonstrated that Uev1A-Ubc13 represses stress-induced apoptosis in HepG2 cells [20] and promotes breast and colon cancer metastasis through the NF-κB signaling pathway [19, 21]. Meanwhile, Uev1A-Ubc13 promotes breast cancer cell survival and chemoresistance through the AKT pathway [6]. Consistently, chemical inhibition of the Uev1A-Ubc13 interaction suppresses cells survival and proliferation of diffuse large B-cell lymphoma cells [22]. These results collectively indicate that Uev1A is involved in tumorigenesis and metastasis.

The PI3K/AKT signaling pathway is an essential node in mammalian cells and is closely associated with various biological functions including cell growth, survival, proliferation, migration, resistance to apopotosis, differentiation, metabolism and angiogenesis [23–26]. In addition, this pathway is frequently found to be abnormally activated and altered in many human malignancies, which induces chemoresistance and malignant transformation [27–30]. AKT has three isoforms, AKT1, AKT2 and AKT3, with highly conserved domain structure [31], which are associated with breast cancer progression and play different roles in breast cancers [32].

Epithelial-mesenchymal transition (EMT) is closely associated with cancer progression, cancer cell metastasis and drug resistance [33, 34]. Cells undergoing EMT display increased expression of mesenchymal genes including *N-cadherin*, *fibronectin* and *vimentin*, and decreased expression of epithelial genes including *E-cadherin*, *occulin* and *ZO-1* [35].

In this study we found that overexpression of *UEV1A* induced *CT45A* expression in breast cancer cells in a Ubc13-dependent manner, while depletion of Uev1 inhibited *CT45A* expression. CTAs are tumor associated and testis-derived specific immunogenic antigens closely associated with spontaneous immune responses in cancer patients [36, 37]. They are not expressed in nearly all normal tissues except testis after birth, but are expressed in various types of cancers [38–43]. The *CT45A* family genes are widely expressed in various malignant cancers and closely associated with tumorigenesis [44], poor prognosis, metastasis and aggressiveness [45–51]. Ectopic expression of *CT45A1* could promote tumorigenesis and metastasis of breast cancer [52], but the underlying mechanism remains unclear. This study revealed that ectopic expression of *CT45A* upregulated expression of its downstream genes related to tumorigensis, EMT, stemness and metastasis, and promoted breast cancer EMT signaling and cell migration. A series of experimental results support a notion that *CT45A* is a critical downstream gene of the AKT but not the NF-κB signaling pathway. Since similar effects were also observed in a colorectal cancer cell line, the Uev1A/Ubc13-AKT-CT45A axis in tumorigenesis may occur in other tissues. Hence, this study suggests a potential therapeutic target in the treatment of breast and colorectal cancers.

## Methods

### Cell lines and culture

Human breast cancer cell lines MCF7 and MDA-MB-231, and human colon carcinoma cell line HCT116 were obtained from the American Type Culture Collection (ATCC, Manassan, VA, USA). The cells were cultured in Dubecco’s modified Eagle medium (DMEM, HyClone), supplemented with 10% fetal bovine serum (FBS, HyClone), 100 units/mL penicillin, and 100 μg/mL streptomycin (Invitrogen) at 37 °C with 5% CO_2_. *UEV1A*-overexpressed stable MDA-MB-231 and MCF7 cell lines were created as previously reported [6]. Stable Uev1A-knockdown cell lines were created by transfecting MDA-MB-231 and MCF7 cells with Uev1A shRNA lentiviral particles or negative control shRNA lentiviral particles-A (Santa Cruz Biotechnology, Inc), and selecting with 1 μg/mL puromycin dihydrochloride (Santa Cruz Biotechnology, Inc).

### Plasmids and cell transfection

Human *UEV1A* and *CT45A* open reading frames (ORFs) were amplified as *Kpn*I-*Xho*I fragments and cloned into a plasmid vector pcDNA4.0/TO/HA (**+**) (Invitrogen) as previously described [19]. The mutated Ubc13-binding site (F38E) in *UEV1A* was designed based on a previous study with Mms2-F13E [17], and Uev1A-F38E is known to abolish physical interaction with Ubc13 [7, 19]. The *CT45A* and *AKT1* small interfering RNAs (siRNAs) were purchased from GenePharma (Shanghai, China). The sequence for *CT45A* siRNA is 5′-GGAGAGAAAAGGAUCAGAUUU-3′ and the sequence for *AKT1* siRNA is 5′-AGGAAGUCAUCGUGGCCAATT-3′. The modified sequence for *UEV1A* small hairpin RNA (shRNA, sc-38606-v) and negative control shRNA (sc-108080) delivered by lentiviral particles were obtained from Santa Cruz Biotechnology, Inc. The lentiviral particle infection of MDA-MB-231 and MCF7 breast cancer cells was performed following instructions of the supplier. The transient transfection of plasmids and siRNAs took 48 or 72 h, respectively.

### RNA preparation and quantitative real-time RT-PCR (qRT-PCR)

Total RNAs were extracted from cultured MDA-MB-231, MCF7 breast cancer and HCT116 colorectal cancer cells using Trizol (Invitrogen, 15596018). First-strand cDNA was synthesized with 1 μg of total RNAs with TransScript® All-in-One First-Strand cDNA Synthesis SuperMix (TransGen, AT341-01) according to manufacturer’s instructions. qRT-PCR analysis based on SYBR® Premix Ex Taq™ (Takara, RR420A) was performed on the BioRad CFX96 real-time PCR machine. The data analysis was performed using the 2^-ΔCT^ comparative cycle threshold method [53] from three independent experiments, with *GAPDH* transcript as an internal reference. Gene-specific primers are listed in Supplemental Table S1.

### Microarray analysis

Plasmids pcDNA4.0/TO/HA-UEV1A and pcDNA4.0/TO/HA vector control (CK) were transfected into MDA-MB-231 cells [19] to create inducable stable cell lines. After 10 μg/mL doxycycline (Dox) treatment, total RNAs were extracted from MDA-MB-231-UEV1A and -CK cells. mRNA quality was assessed by electrophoresis of total RNA followed by staining with ethidium bromide, and the 2:1 ratio of 28S:18S indicated high quality RNA to be used for the microarray experiment. Microarray (Roche Nimblegen Human 12x135K) were analyzed by Capitalbio Croppration, Beijing, China. Each sample was measured in duplicate. Compared with the vector control, all genes with altered expression in the *UEV1A*-overexpressed group were identified.

### Protein extraction and western blotting

Cells were grown to log phase and lysed with a whole-cell extraction buffer (150 mM NaCl, 1% NP-40, 10% glycerol, 1 mM EDTA, 50 mM Tris, 1 mM PMSF) and protease inhibitor cocktail for mammalian cells (Roche). Proteins in cell extracts were separated by 8–12% SDS-PAGE gels and transferred to PVDF membrane. The membrane was blocked with 5% milk/BSA and incubated with specific primary antibodies followed by secondary antibodies. The following antibodies were used: anti-AKT (#4691, Cell Signaling Technology, 1:1000), anti-Phospho-AKT-Ser473 (#4060, Cell Signaling, 1:1000), anti-Phospho-AKT-Thr308 (#13038, Cell Signaling, 1:1000), anti-Tubulin (sc-166729, Santa Cruz, 1:5000), anti-HA (A-190-208A, Bethyl 1:2500), anti-N-cadherin (#13116, Cell Signaling, 1:500), anti-E-cadherin (#3195S, Cell Signaling, 1:500), anti-Lamin B (sc-6216, Santa Cruz, 1:500), anti-NF-κB p65 (sc-8008, Santa Cruz 1:100), anti-CT45A (SAB1301842, Sigma, 1:1000), goat anti-mouse IgG-horseradish peroxidase (HRP) (sc-2005, Santa Cruz, 1:5000), goat anti-rabbit IgG-HRP (sc-2004, Santa Cruz, 1:5000) and donkey anti-goat IgG-HRP (sc-2033, Santa, Cruz, 1:5000). The following inhibitors were used: NF-κB pathway inhibitor Bay117082 (tlrl-b82, Invivogen, 30 μM for 24 h), AKT pathway inhibitor LY294002 (Selleck, 10 μM for 24 h), IGF-1 (HY-P7018, Medchem Express, 100 ng/mL for 1–3 h).

### Nuclear fraction preparation

MDA-MB-231, MCF7 and HCT116 cells were grown to log phase and lysed with buffer A (10 mM HEPES, 10 mM KCl, 0.34 M sucrose, 1 mM DDT, 10% glycerol, 1.5 mM MgCl_2_, 0.1% Triton X100, 1 mM PMSF) and the protease inhibitor cocktail for mammalian cells (Roche), incubated on ice for 5 min and centrifuged at 4000 rpm for 4 min at 4 °C. After the cytosolic supernatant was transferred to a new tube, the pellet was resuspended in a whole-cell extraction buffer (150 mM NaCl, 1% NP-40, 10% glycerol, 1 mM EDTA, 50 mM Tris, 1 mM PMSF) and the protease inhibitor cocktail for mammalian cells (Roche), stored on ice for 30 min, followed by centrifugation at 13,200 rpm for 15 min at 4 °C. The supernatant was collected as the nuclear fraction.

### Cell migration assay

In vitro cell migration ability was measured by a Transwell assay without Matrigel coating, using 8-μm-pore-size polycarbonate membrane filters in 24-well culture plates. Briefly, after incubation for 16–18 h, cells were transfected with indicated plasmids. 10–12 h later, cells were starved in FBS-free DMEM medium for 12–14 h, and then 2 **×** 10^5^ HCT116, 5 **×** 10^4^ MDA-MB-231 or 2 **×** 10^5^ MCF7 cells were seeded in the upper chamber, while the lower surface of the filter was coated with 10% FBS-DMEM as chemo-attractants. The cells were allowed to migrate for 24 h and those migrated to the lower surface of the filter were counted in five random fields under a light-microscope at high magnification. These experiments were done at least in triplicate.

### Statistical analysis

The statistical significance of differential findings between the control and experimental groups was determined by student’s t-test as implemented by Microsoft Excel 2016 (*, *P* < 0.05; ** *P* < 0.01; and ***, *P* < 0.001).

## Results

### Uev1A upregulates *CT45A* expression in a Ubc13-dependent manner

**Fig. 1:**
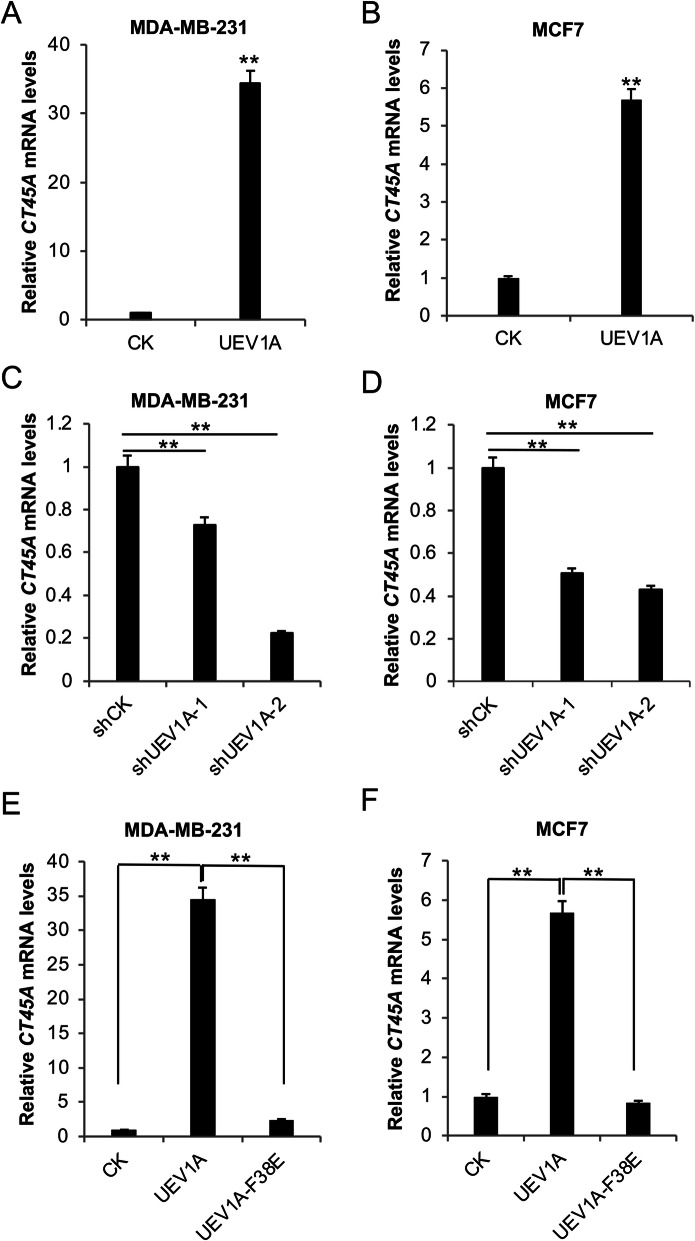
Uev1A upregulates *CT45A* expression in a Ubc13-dependent manner in breast cancer cells. **a, b** Relative *CT45A* transcript levels in *UEV1A*-overexpressed MDA-MB-231 (**a**) and MCF7 (**b**) cell lines were monitored by qRT-PCR. **c, d** MDA-MB-231 (**c**) and MCF7 (**d**) cells were transfected with shRNA lentiviral particles against *UEV1A* (shUEV1A) or non-specific target (shCK). shUEV1A-1 and shUEV1A-2 represent two independent stable shUEV1A cell lines. *CT45A* transcript levels in shCK and shUEV1A cell lines were monitored by qRT-PCR. **e, f** Overexpressed *UEV1A* but not *UEV1A-F38E* upregulated *CT45A* expression in MDA-MB-231 (**e**) and MCF7 (**f**) cells, as determined by qRT-PCR. CK, control treatment. All experiments were performed in at least triplicate and the results are the average with standard deviation. **, *P* < 0.01

We performed a microarray analysis by comparing *UEV1A*-overexpressed and vector control MDA-MB-231 breast cancer cells, which revealed 47 genes upregulated by more than fivefold in *UEV1A*-overexpressed MDA-MB-231 cells (Supplemental Table S2). Interestingly, 16 out of 47 belong to cancer/testis antigens (CTAs), among which *CT45A* family members are most highly elevated in *UEV1A*-overexpressed MDA-MB-231 cells (Supplemental Fig. S1a). The *CT45A* gene family comprises 10 genes designated as *CT45A1* to *CT45A10*, which are distinct but highly conserved, as their amino-acid sequences exhibit more than 98% identity [54] (Fig. S1b). Our attempt to detect endogenous CT45A proteins in several cancer cell lines including those used in this study was unsucessful (Fig. S2), although the same commercial polyclonal antibody has been used to study CT45A in ovarian cancer cells [54]. To independently examine the role of Uev1A in upregulating *CT45A* expression and its biological implications, *UEV1A* was cloned into a pcDNA4.0/TO/HA(**+**) vector and then transiently trasfected into MDA-MB-231 and MCF7 cells. The level of *UEV1A* ectopic expression was monitored by western blot against the HA-tag (Fig. S3a, b). Then *CT45A* expression was measured by qRT-PCR and found to be signifcantly upregulated in *UEV1A*-overexpressed MDA-MB-231 (Fig. 1a) and MCF7 (Fig. 1b) cells. It has been reported that *UEV1A* is upregulated in MDA-MB-231 and MCF7 cells by 2.8- and 4-fold, respectively [19]. To ask whether this moderate overexpression of *UEV1A* contributes to *CT45A* upregulation in breast cancer cells, we depleted endogenous Uev1A in MDA-MB-231 and MCF7 cells using shRNAs delivered by lentiviral particles as previously reported [19]. It was found that two independent shUEV1A constructs, shUEV1A-1 and shUEV1A-2, reduced *UEV1A* transcript levels in MDA-MB-231 cells by 43 and 60% (Fig. S4a), and in MCF7 cells by 71 and 85% (Fig. S4b), respectively, compared to contral shRNA-treated cells. Meanwhile, *CT45A* transcript levels were also reduced (Fig. 1c, d). To further ask whether Uev1A upregulates *CT45A* expression in a Ubc13-dependent manner, we transiently transfected MDA-MB-231 and MCF7 cells with a construct encoding the Uev1A-F38E mutant protein (Fig. S3a, b). As expected, Uev1A-F38E failed to upregulate *CT45A* mRNA levels in both MDA-MB-231 (Fig. 1e) and MCF7 (Fig. 1f) cells. These observations collectively indicate that Uev1A upregulates *CT45A* expression in a Ubc13-dependent manner in breast cancer cells.

### Uev1A positively regulates *CT45A* downstream gene expression in breast cancer cells

**Fig. 2:**
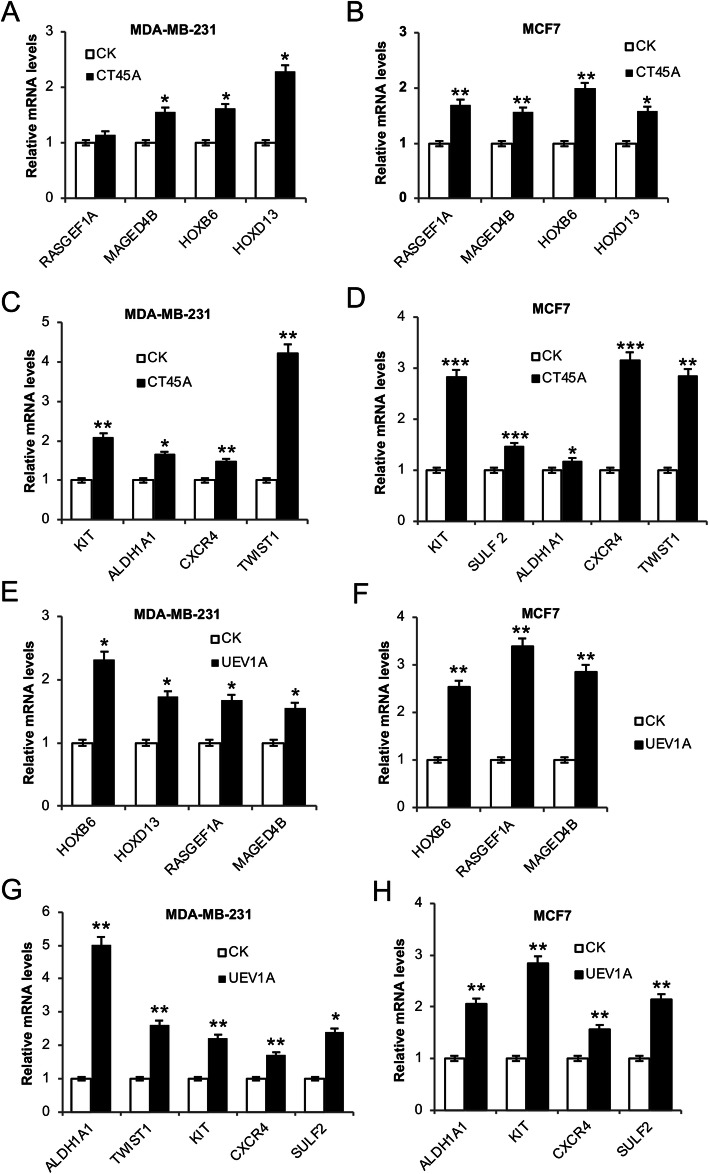
Uev1A positively regulates expression of *CT45A* downstream genes in breast cancer cells. **a, b** Transcript levels of tumorigenic genes, including *RASGEF1A*, *MAGED4B*, *HOXB6* and *HOXD13* in *CT45A*-overexpressed MDA-MB-231 (**a**) and MCF7 (**b**) cells were detected by qRT-PCR. **c, d** Transcript levels of EMT, stemness and metastatic genes, including *KIT*, *ALDH1A1*, *CXCR4*, *TWIST1* and/or *SULF2* in *CT45A*-overexpressed MDA-MB-231 (**c**) and MCF7 (**d**) cells were detected by qRT-PCR. **e, f** Expression of *CT45A* downstream tumorigenic genes, including *HOXB6*, *RASGEF1A*, *MAGED4B* and/or *HOXD13* in *UEV1A* transiently overexpressed MDA-MB-231 (**e**) and MCF7 (**f**) cells were monitored by qRT-PCR. **g, h** Expression of *CT45A* downsream EMT, stemness and metastasic genes, including *ALDH1A1*, *KIT*, *CXCR4*, *SULF2* and/or *TWIST1* were monitored in *UEV1A* transiently overexpressed MDA-MB-231 (**g**) and MCF7 (**h**) cells by qRT-PCR. CK, control treatment. All experiments were performed in at least triplicate and the results are the average with standard deviation. *, *P* < 0.05; **, *P* < 0.01; and ***, *P* < 0.001

*CT45A* has been reported to act as a proto-oncogene through upregulating tumorigenic and metastatic genes [52]. We first measured the transcript level of several previously-reported [52] *CT45A* downstream genes thought to be involved in tumoregenesis, EMT, stemness and metastasis after *CT45A* ectopic expression. The expression of some tumoregenesis-associated genes, including those encoding RAS exchange factor (*RASGEF1A*), melanoma antigen family member (*MAGED4B*), homeobox B6 (*HOXB6* and *HOXD13*) was indeed significantly higher in *CT45A*-overexpressed MDA-MB-231 (Fig. 2a) and MCF7 (Fig. 2b) cells than their respective control cells. Expression of several EMT, stemness and metastasis related genes, including *TWIST1*, *KIT*, aldehyde dehydrogenase 1 family member A1 (*ALDH1A1*), *CXCR4* and *SULF2* was also upregulated in *CT45A*-overexpressed MDA-MB-231 (Fig. 2c) and MCF7 (Fig. 2d) cells. Since Uev1A can upregulate *CT45A* expression, we asked whether Uev1A could also upregulate the expression of *CT45A* downstream genes in breast cancer cells. Indeed, the majority of *CT45A* downstream genes, including *HOXB6, HOXD13, RASGEF1A*, *MAGED4B*, *ALDH1A1*, *TWIST1*, *KIT*, *CXCR4* and *SULF2*, were upregulated in *UEV1A*-overexpressed MDA-MB-231 (Fig. 2e, g) and MCF7 (Fig. 2f, h) cells. Taken together, we conclude that Uev1A positively regulates *CT45A* downstream gene expression in breast cancer cells.

### CT45A is a critical regulator for Uev1A-induced breast cancer cell migration

**Fig. 3:**
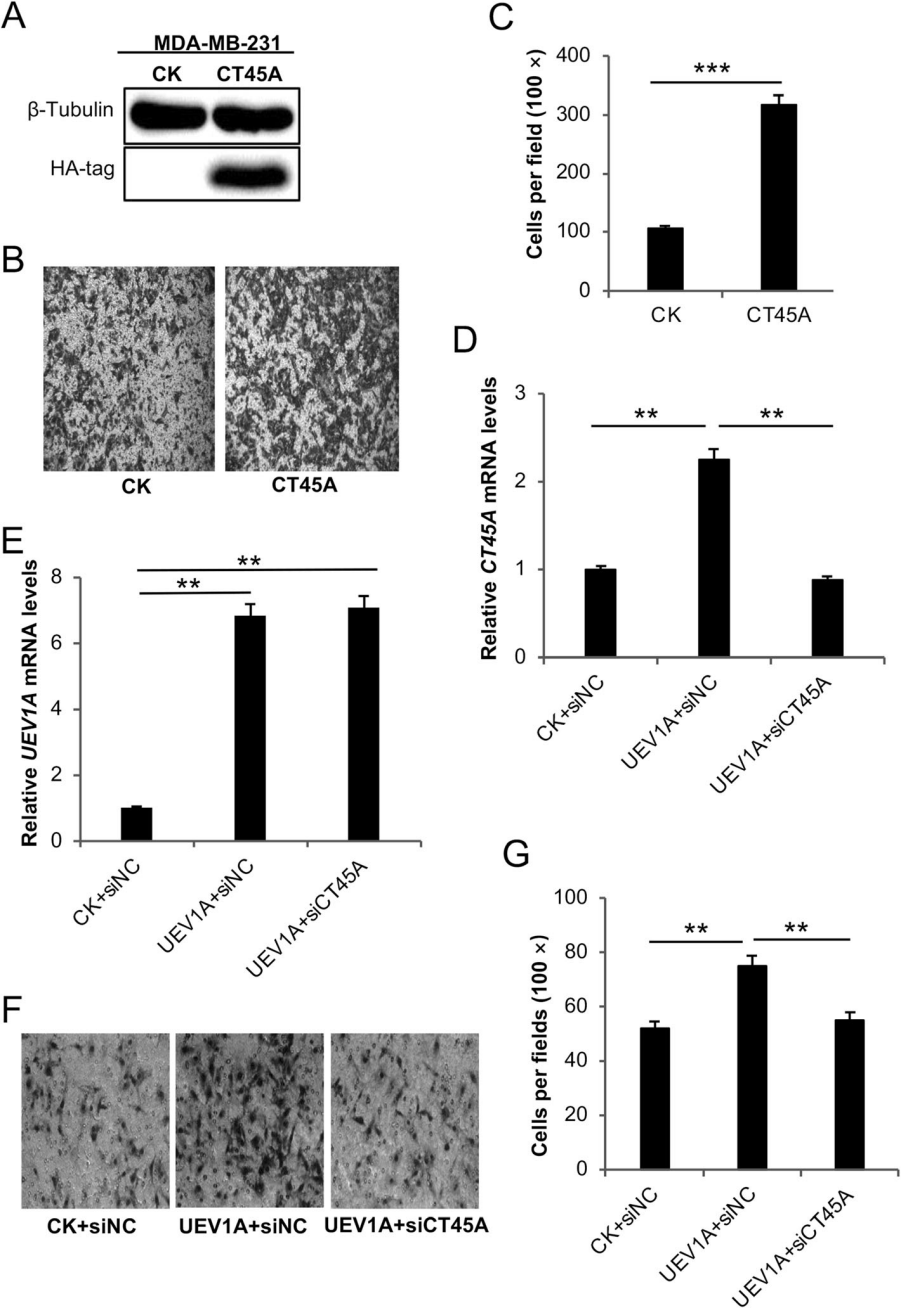
Effects of altered *UEV1A* and *CT45A* expression on MDA-MB-231 cell migration. **a** The ectopic *CT45A* expression was monitored by western blot against an HA-tag antibody. The gel images are cropped from available original blots. **b** Representative images of cell migration using the transwell assay. **c** Statistical analysis of the cell migration assay data. Cells migrated to the lower surface of the filter were counted in five random fields under a light-microscope at 100 × magnification. **d, e** The relative expression of *CT45A* (**d**) and *UEV1A* (**e**) in CT45A-depleted and *UEV1A* transiently overexpressed cells was monitored by qRT-PCR. **f** Representative images of cell migration ability using the transwell assay. MDA-MB-231 cells transiently expressing *UEV1A* were depleted of CT45A and subjected to the transwell assay. **g** Statistical analysis of the cell migration assay data. Cells that migrated to the lower surface of the filter were counted in five random fields under a light-microscope at 100 × magnification. CK, control treatment; siNC, control siRNA. All experiments were performed in at least triplicate and the results are the average with standard deviation. **, *P* < 0.01; and ***, *P* < 0.001

**Fig. 4:**
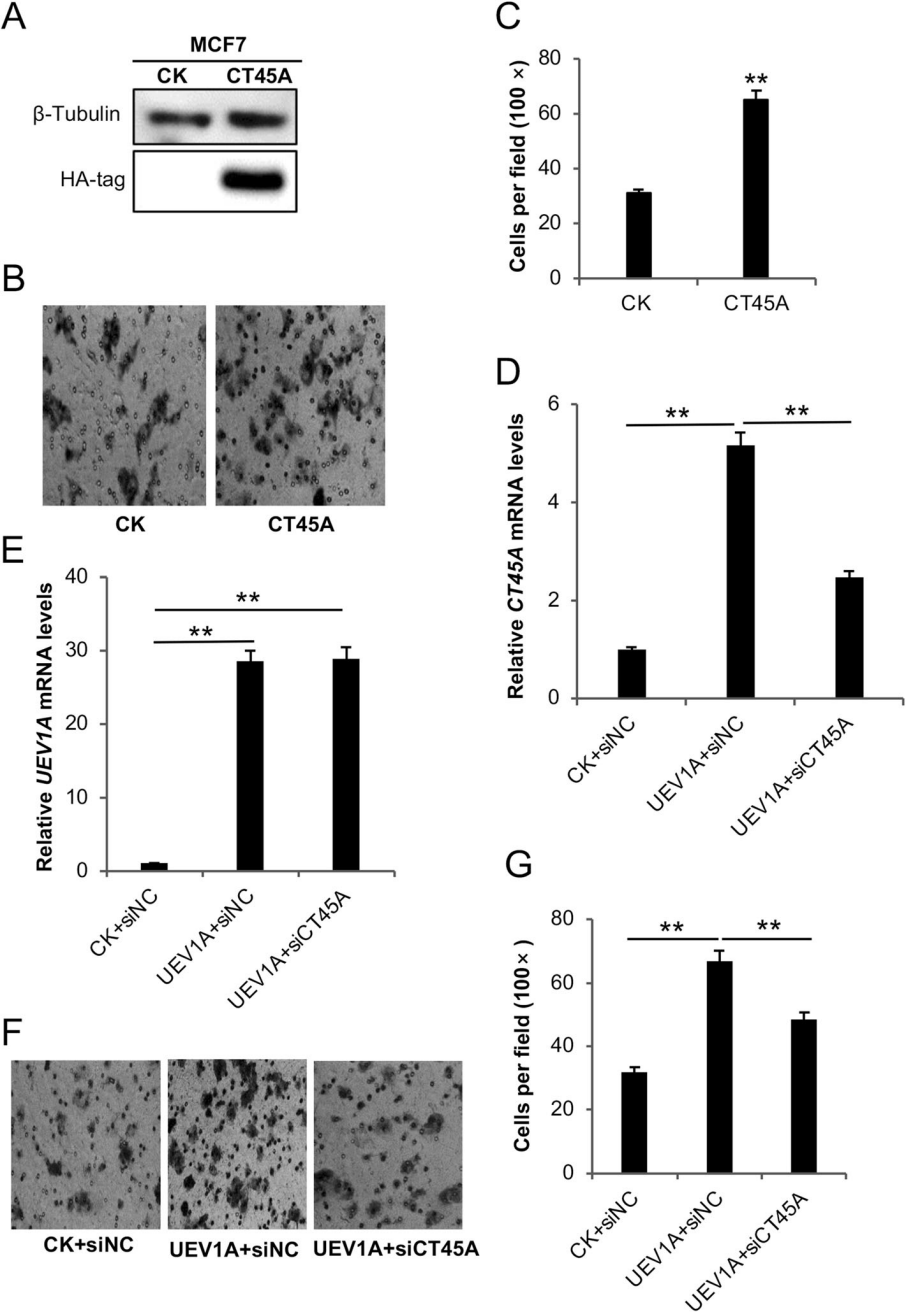
Effects of altered *UEV1A* and *CT45A* expression on MCF7 cell migration. **a** The ectopic *CT45A* expression was monitored by western blot against an HA-tag antibody. The gel images are cropped from available original blots. **b** Representative images of cell migration using the transwell assay. **c** Statistical analysis of the cell migration assay data. **d, e** The relative expression of *CT45A* (**d**) and *UEV1A* (**e**) in CT45A-depleted and *UEV1A* transiently overexpressed cells was monitored by qRT-PCR. **f** Representative images of cell migration ability using the transwell assay. MCF7 cells transiently expressing *UEV1A* were depleted with CT45A and subjected to the transwell assay. **g** Statistical analysis of the cell migration assay data. Cells that migrated to the lower surface of the filter were counted in five random fields under a light-microscope at 100 × magnification. CK, control treatment; siNC, control siRNA. All experiments were performed in at least triplicate and the results are the average with standard deviation. **, *P* < 0.01

To ask whether an elevated *CT45A* level alone is indeed sufficient to promote breast cancer cell migration, *CT45A* was cloned into plasmid pcDNA4.0/TO/HA(**+**), transiently transfected into MDA-MB-231 and MCF7 cells and the level of *CT45A* ectopic expression after treatment with 200 μg/mL zeocin was monitored by western blot against an HA-tag antibody (Figs. 3a and 4a). The effects of *CT45A* ectopic expression on MDA-MB-231 (Fig. 3) and MCF7 (Fig. 4) cells were then assessed. The transwell experiments showed that overexpression of *CT45A* increased the MDA-MB-231 cell mobility by nearly threefold compared with vector-transfected cells (Fig. 3b, c). Similarly, after selection with zeocin, the migration of MCF7 *CT45A* transfectants was 2.3-fold higher than the control cells (Fig. 4b, c), indicating that CT45A regulates breast cancer cell migration in vitro.

To ask whether Uev1A is a critical regulator for CT45A-induced migration, we successfully depleted CT45A by approximately 50% using siRNA in MDA-MB-231 (Fig. S4c) and MCF7 (Fig. S4d), as well as *UEV1A*-overexpressed MDA-MB-231 (Fig. 3d) and MCF7 (Fig. 4d) cells. The above treatment does not affect the expression of *UEV1A* (Figs. 3e and 4e), but the moderate CT45A depletion in *UEV1A*-overexpressed cells markedly reduced cell migration as determined by a transwell assay (Figs. 3f, g and 4f, g). The above findings allow us to conclude that CT45A is a critical regulator for Uev1A-induced migration in breast cancer cells, as partial depletion of CT45A can reverse cell migration in *UEV1A*-overexpressed breast cancer cells.

### CT45A promotes cell migration of HCT116 colorectal cancer cells

**Fig. 5:**
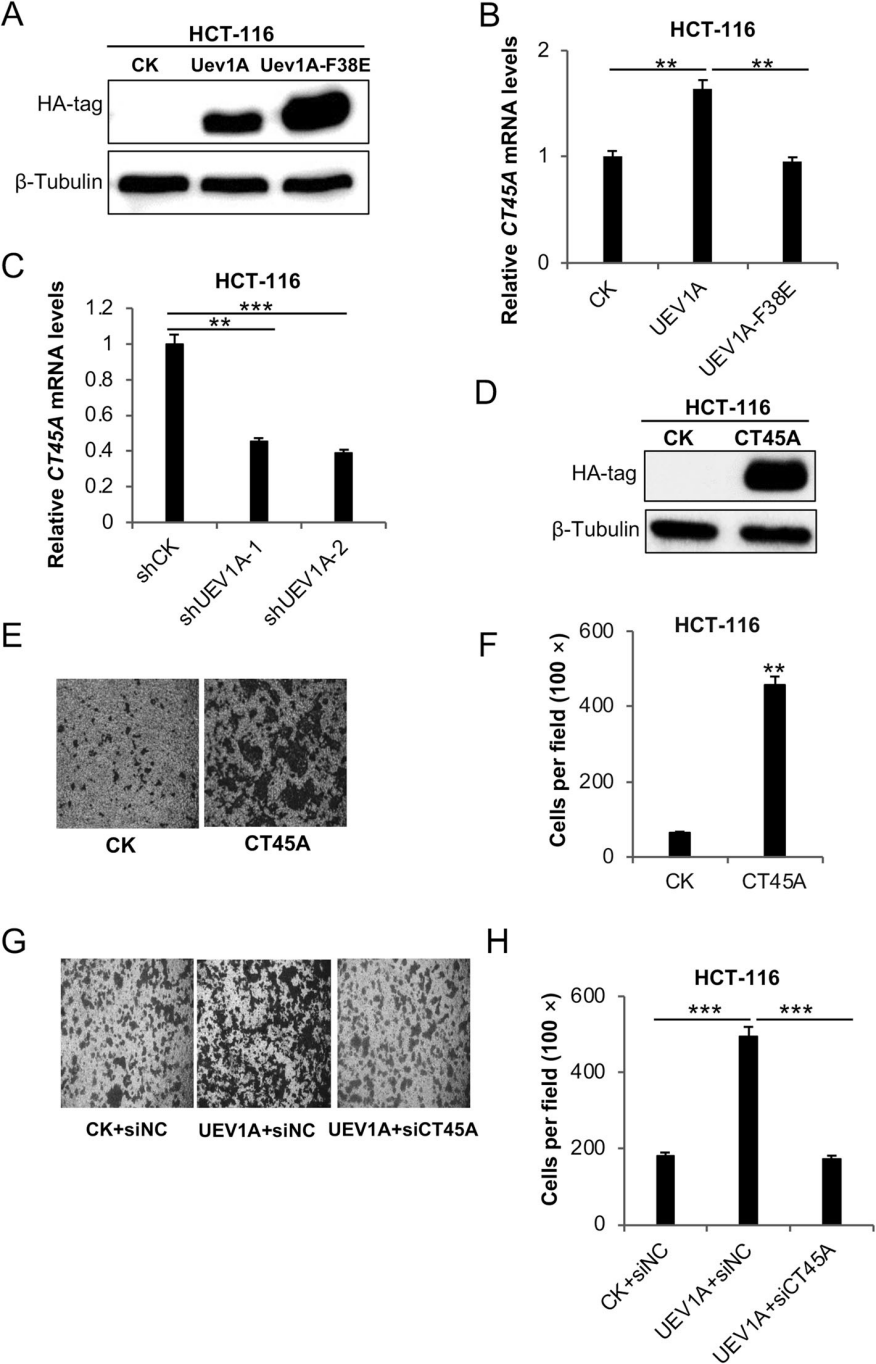
CT45A promotes cell migration of HCT116 colorectal cancer cells. **a** Cellular HA-tagged Uev1A and Uev1A-F38E were detected by western blot against an anti-HA antibody. **b** Overexpressed *UEV1A* but not *UEV1A-F38E* upregulated *CT45A* expression in HCT116 cells. **c** HCT116 cells were transfected with shRNA lentiviral particles against *UEV1A* (shUEV1A) or non-specific target (shCK). *CT45A* transcript levels in shCK and shUEV1A cell lines were monitored by qRT-PCR. **d** Cellular HA-tagged CT45A was detected by western blot against an anti-HA antibody. **e** Representative images of HCT116 cell migration using the transwell assay. **f** Statistical analysis of the cell migration assay data. Cells that migrated to the lower surface of the filter were counted in five random fields under a light-microscope at 100 × magnification. **g** Representative images of HCT116 cell migration ability using the transwell assay. HCT116 cells expressing *UEV1A* were depleted of CT45A and subjected to the transwell assay. **h** Statistical analysis of the cell migration assay data. CK, control treatment; siNC, control siRNA. All experiments were performed in at least triplicate and the results are the average with standard deviation. **, *P* < 0.01; and ***, *P* < 0.001. The gel images in (**a**) and (**d**) are cropped from available original blots

To ask whether *UEV1A* overexpression also increases *CT45A* expression in other cancer cells, we transiently transfected *UEV1A* in HCT116 colorectal cancer cell lines (Fig. 5a), in which *CT45A* was moderately upregulated upon *UEV1A* ectopic expression, but not in *UEV1A*-*F38E*-expressed HCT116 cells (Fig. 5b). To ask whether the moderate elevation of Uev1A contributes to *CT45A* upregulation in colorectal cancer cells, we depleted endogenous Uev1A in HCT116 cells by using shRNAs delivered by lentiviral particles as previously reported [21]. It was found that two independent shUEV1A constructs, shUEV1A-1 and shUEV1A-2, reduced *UEV1A* transcript levels by 55 and 65%, respectively, in HCT116 cells compared to control shRNA-treated cells (Supplemental Fig. S5a). Meanwhile, *CT45A* transcript levels were also reduced (Fig. 5c). To ask whether ectopic expression of *CT45A* could promote metastasis signaling in colorectal cancer cells, HCT116 cells were transiently transfected with pcDNA4.0/TO/HA-CT45A and the CT45A level was monitored by western blot analysis against HA-tagged CT45A (Fig. 5d). The *CT45A* ectopic expression resulted in concomitant increase in HCT116 cell migration by sevenfold (Fig. 5e, f), indicating that CT45A could also promote cell migration in colorectal cancer cells. To further ask whether CT45A is a critical regulator for Uev1A-induced migration, we depleted CT45A by using siRNA in *UEV1A*-overexpressed HCT116 cells. As shown in Fig. S5b, *CT45A* was depleted by 44%. The above treatment does not affect the expression of *UEV1A* (Fig. S5c), but the moderate CT45A depletion in *UEV1A*-overexpressed HCT116 cells markedly reduced cell migration as determined by a transwell assay (Fig. 5g, h). The above findings indicate that Uev1A induces colorectal cancer cell migration through upregulating *CT45A* genes.

### Depletion of *CT45A* reverses EMT signaling in *UEV1A*-overexpressed breast cancer cells

**Fig. 6:**
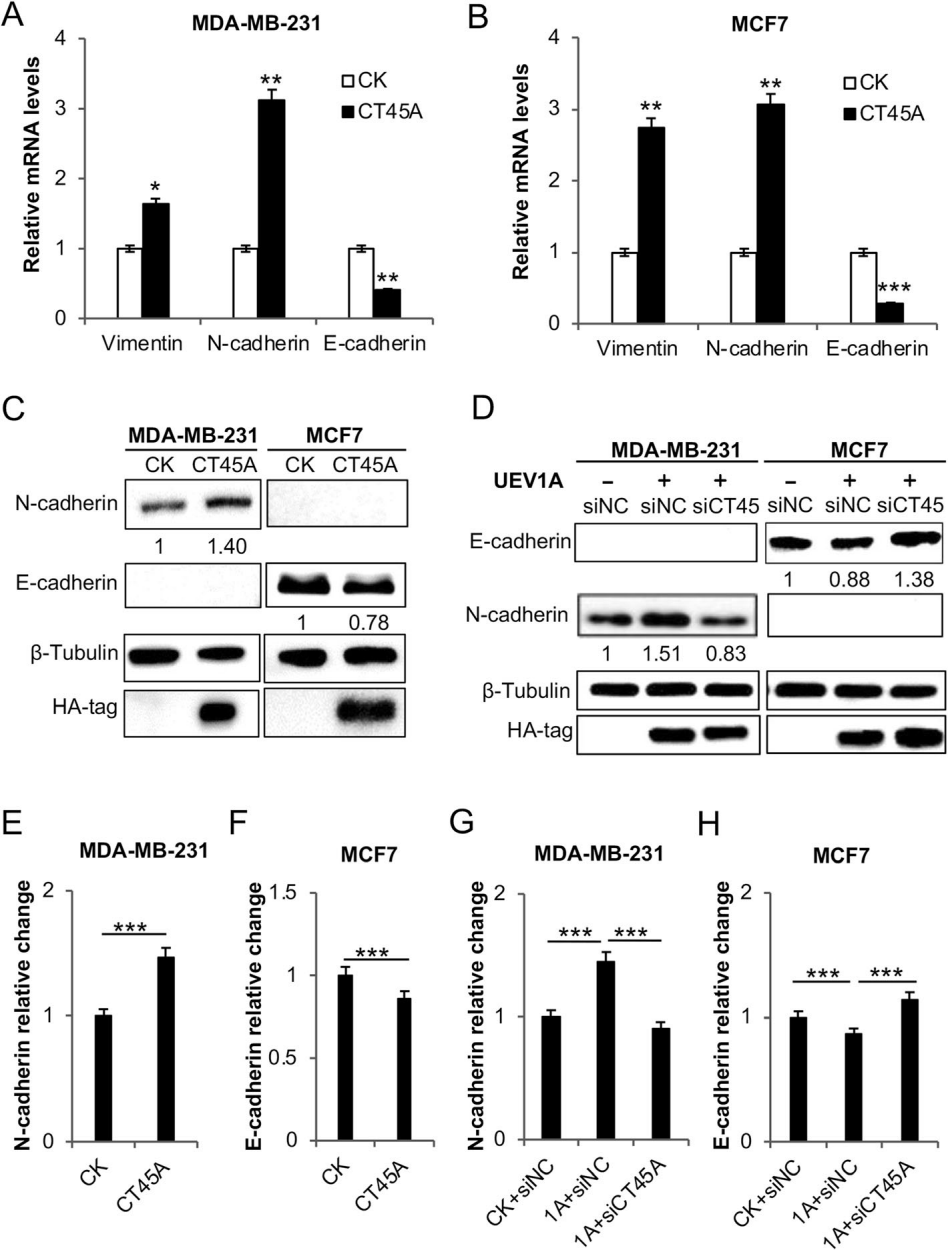
Effects of *CT45A* and *UEV1A* expression on EMT signaling in breast cancer cells. **a, b** Relative transcript levels of EMT markers, including epithelial markers *E-cadherin* and mesenchymal markers *N-cadherin*, *vimentin* in *CT45A*-overexpressed MDA-MB-231 (**a**) and MCF7 (**b**) cells were determined by qRT-PCR. **c** The expression of N-cadherin and E-cadherin in *CT45A*-overexpressed MDA-MB-231 (left panel) and MCF7 (right panel) cells was detected by western blot against anti-N-cadherin and anti-E-cadherin antibodies. **d** The expression of N-cadherin and E-cadherin in MDA-MB-231 (left panel) and MCF7 (right panel) cells transiently overexpressing *UEV1A* and depleted of CT45A was detected by western blot using anti-N-cadherin and anti-E-cadherin antibodies. The gel images in (**c**) and (**d**) are cropped from available original blots. Numbers underneath the WB images indicate relative band intensity after normalization with the loading control. **e-h** Statistical analyses of relative cellular N-cadherin and E-cadherin levels in MDA-MB-231 (**e, g**) and MCF7 (**f, h**) cells as indicated in the graphs. CK, control treatment; siNC, control siRNA. All experiments were performed in at least triplicate and the results are the average with standard deviation. **, *P* < 0.01; and ***, *P* < 0.001

It was reported that overexpression of *CT45A* could induce breast cancer EMT, and thus foster cancer metastasis by upregulating EMT master gene *TWIST1* [52]. To further investigate the potential molecular mechanism by which *CT45A* regulates breast cancer cell migration, we monitored the alteration of EMT markers, including *N-cadherin* and *vimentin*, two well-characterized mesenchymal markers, and *E-cadherin*, a well-known epithelial marker [35, 55]. It was to our surprise that the *N-cadherin* mRNA level in MDA-MB-231 was nearly 90-fold higher than in MCF7 (Fig. S6a), while the *E-cadherin* mRNA level in MCF7 was nearly 50-fold higher than in MDA-MB-231 (Fig. S6b). Consistent with breast cancer cell migration, increased mRNA levels of *N-cadherin* and *vimentin* and decreased *E-cadherin* were found upon *CT45A* overexpression in MDA-MB-231 (Fig. 6a) and MCF7 (Fig. 6b) cells. We also assessed effects of CT45A on cellular N-cadherin and E-cadherin at protein levels. Firstly, we monitored cellular N-cadherin and E-cadherin levels in MDA-MB-231 and MCF7 cells and found that, consistently with their corresponding transcript levels in the two cell lines (Fig. S6), MDA-MB-231 cells only produced detectable N-cadherin, while MCF7 cells only produced detectable E-cadherin (Fig. 6c). Interestingly, ectopic expression of *CT45A* increased N-cadherin in MDA-MB-231 cells and decreased E-cadherin in MCF7 cells (Fig. 6c, e, f), suggesting that cell migration stimulated by ectopic *CT45A* expression was likely due to the enhanced EMT in breast cancer cells. To address whether Uev1A is a critical upstream regulator of CT45A-induced EMT signaling, we depleted CT45A by using siRNA in *UEV1A*-overexpressed MDA-MB-231 and MCF7 breast cancer cells (Figs. 3d and 4d), which significantly increased E-cadherin protein levels in *UEV1A*-overexpressed MCF7 cells and decreased N-cadherin protein levels in *UEV1A*-overexpressed MDA-MB-231 cells (Fig. 6d, g, h). Collectively, these results support a notion that Uev1A can serve as an important regulator for CT45A-induced EMT signaling in breast cancer cells.

### Uev1A regulates *CT45A* expression through the AKT signaling pathway

**Fig. 7:**
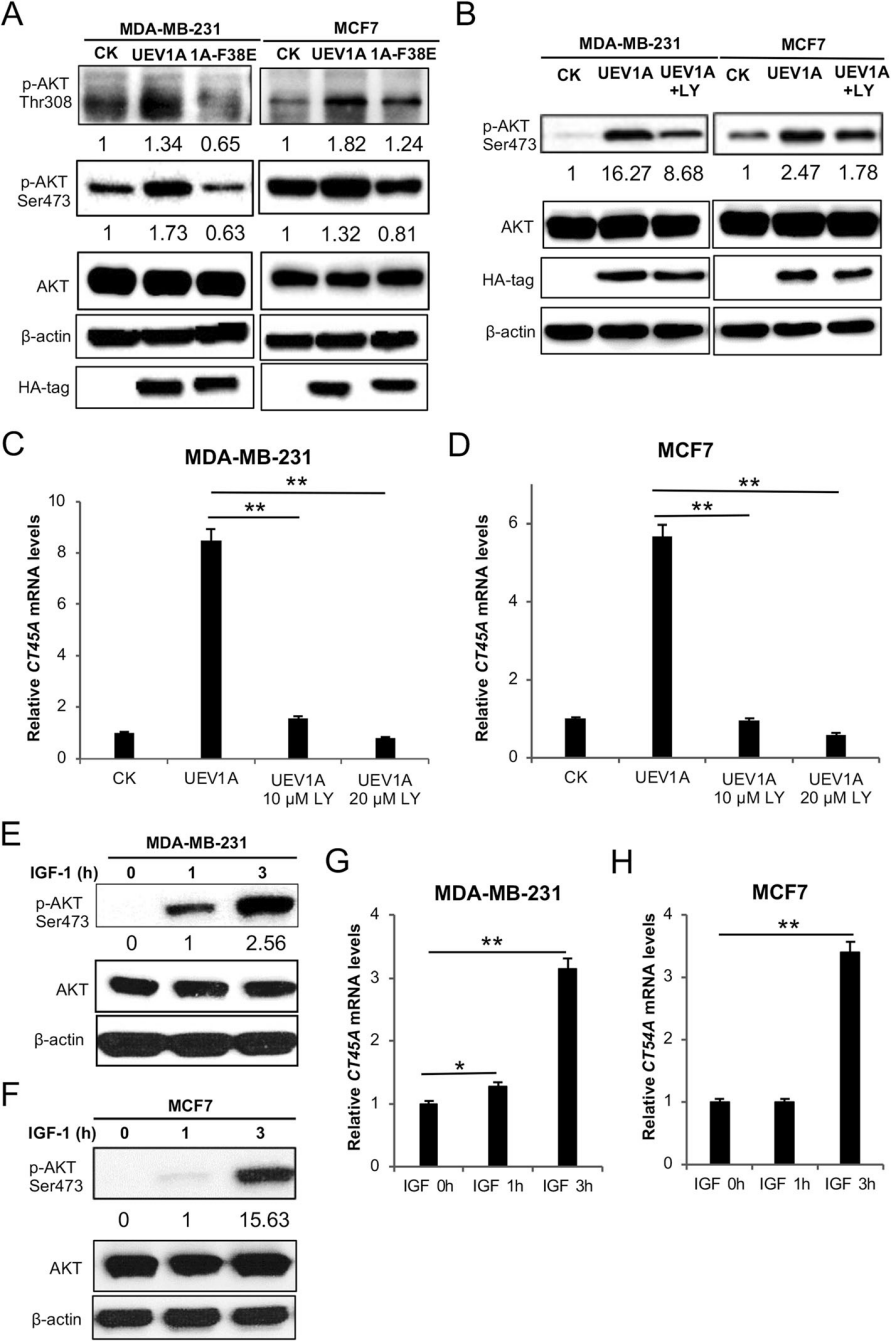
Uev1A regulates *CT45A* expression through the AKT signaling pathway. **a** The cellular AKT protein and its phosphorylation (p-AKT-Thr308, p-AKT-Ser473) levels in pcDNA4.0/TO/HA (+) vector (CK), *UEV1A*, *UEV1A-F38E* transiently transfected MDA-MB-231 (left panel) and MCF7 (right panel) cells were monitored by western blot using anti-AKT, anti-p-AKT-Thr308 and anti-p-AKT-Ser473 antibodies. **b** The *UEV1A* transiently transfected MDA-MB-231 (left panel) and MCF7 (right panel) cells were treated with 10 μM PI3K/AKT pathway inhibitor LY294002. After 24 h, the AKT and p-AKT-Ser473 levels were examined by western blot using anti-AKT, anti-p-Ser473 antibodies in cells transfected with vector, *UEV1A* with or without LY294002 treatment as indicated. Ectopic *UEV1A* expression was detected by an anti-HA antibody. **c, d** Relative *CT45A* expression levels in MDA-MB-231 (**c**) and MCF7 (**d**) cells transfected with vector, *UEV1A* with or without LY294002 treatment as indicated, followed by qRT-PCR. **e, f** MDA-MB-231 (**e**) and MCF7 (**f**) cells were treated with IGF-1 over time as indicated and the cellular AKT and p-AKT-Ser473 proteins were monitored by western blot using anti-AKT and anti-p-AKT-Ser473 antibodies. **g, h** Transcript levels of *CT45A* in MDA-MB-231 (**g**) and MCF7 (**h**) cells treated with IGF-1 over time were monitored by qRT-PCR. CK, control treatment; LY, LY294002. All experiments were performed in at least triplicate and the results are the average with standard deviation. **, *P* < 0.01. **a, b, e, f** The gel images in are cropped from available original blots and numbers underneath the WB images indicate relative band intensity after normalization with the loading control

Since Uev1A has been associated with NF-κB [19–21] and AKT [6] activation, we wish to investigate molecular mechanisms by which Uev1A regulates *CT45A* expression. To ask whether Uev1A regulates *CT45A* expression through the NF-κB pathway, MDA-MB-231, MCF7 and HCT116 cells transiently overexpressing *UEV1A* were treated with the NF-κB pathway inhibitor Bay11-7082 [56] and its efficacy was measured by the nuclear P65 level (Supplemental Fig. S7a-c). The *CT45A* transcript level was not significantly altered in *UEV1A*-ovexpressed MDA-MB-231 (Fig. S7d), MCF7 (Fig. S7e) and HCT116 (Fig. S7f) cells by treatment with Bay11-7082, indicating that Uev1A upregulation of *CT45A* expression is independent of the NF-κB pathway. To ask whether Uev1A regulates *CT45A* expression through the AKT pathway in breast cancer cells, phosphorylation levels of both AKT-Thr308 and AKT-Ser473 in MDA-MB-231 and MCF7 cells transiently overexpressing *UEV1A* were first monitored by western blot and found to be increased (Fig. 7a). In contrast, overexpression of *UEV1A-F38E* failed to induce AKT phosphorylation at both residues (Fig. 7a), indicating that the effects of Uev1A on AKT is dependent on its interaction with Ubc13. These observations allow us to conclude that excessive Uev1A promotes the Uev1A-Ubc13 complex formation, which activates the AKT signaling pathway. To further address whether Uev1A promotes *CT45A* expression through the AKT signaling pathway, we examined effects of PI3K/AKT pathway inhibitor LY294002 [57] on MDA-MB-231 and MCF7 cells with ectopic *UEV1A* expression. As seen in Fig. 7b, the AKT-Ser473 phosphorylation level was markedly decreased after LY294002 treatment in *UEV1A*-overexpressed MDA-MB-231 and MCF7 cells compared to those without the inhibitor treatment. We then examined *CT45A* expression and found that, compared to cells without LY294002 treatment, the *CT45A* transcript level was significantly reduced in *UEV1A*-overexpressed MDA-MB-231 (Fig. 7c) and MCF7 (Fig. 7d) cells after 10 μM LY294002 treatment. After 20 μM LY294002 treatment, the *CT45A* transcript further decreased to levels below the vector control cells without the inhibitor treatment (Fig. 7c, d). It was previously reported that insulin-like growth factor (IGF-1) is an important activator of the PI3K/AKT signaling pathway [58, 59]. To further investigate whether *CT45A* is indeed a direct downstream gene of the AKT signaling pathway, we treated MDA-MB-231 (Fig. 7e) and MCF7 (Fig. 7f) cells with IGF-1, and found that the AKT-Ser473 phosphorylation level was dramatically increased after IGF-1 treatment compared to untreated cells. Under the above experimental conditions, the *CT45A* mRNA levels were significantly increased in MDA-MB-231 (Fig. 7g) and MCF7 (Fig. 7h) cells after IGF-1 treatment. Collectively, we conclude that Uev1A-Ubc13 regulates *CT45A* expression through the AKT signaling pathway in breast cancer cells.

We surveyed relative expression of the three *AKT* genes and found that in both MDA-MB-231 (Fig. S8a) and MCF7 (Fig. S8b) cells, *AKT1* transcript levels were higher than *AKT2*, while the *AKT3* transcript was barely detectable. We depleted AKT1 by using an siRNA and found that the AKT1 levels were reduced by 47% in MDA-MB-231 cells (Fig. S8c) and 35% in MCF7 cells (Fig. S8d) in comparison to control siRNA-transfected cells. Meanwhile, the *CT45A* transcript levels were reduced by 46 and 34% in MDA-MB-231 (Fig. S8e) and MCF7 (Fig. S8f) cells, respectively. The above findings indicate that Uev1A regulates the *CT45A* expression mainly through AKT1.

## Discussion

Previous reports have identified CT45A as a chemosensitivity mediator and immunotherapy target in ovarian cancer [54, 60]. In addition, CT45A has no detectable expression in normal tissues after birth, except for the testis, but it is closely associated with the progression and development of various cancers [44, 45, 52, 61, 62]. In particular, it is highly expressed in cancer stem cells (CSCs), but not in differentiated cells [63], indicating that it is a promising biomarker for diagnosis and treatment of cancer patients. However, exactly how the *CT45A* family genes function in these processes remain unclear.

The *CT45A* family genes were brought to our attention based on our preliminary microarray data from which *CT45A* family genes were among the most highly induced genes following *UEV1A* overexpression in MDA-MB-231 breast cancer cells. This obervation was independently confirmed in two breast cancer cell lines, although the levels of *CT45A* induction after *UEV1A* overexpression vary. In this study, we first investigated the correlation between CT45A and tumorigenesis using breast cancer cell models. At the beginning of our investigation, the *CT45A* gene family was thought to comprise six members (*CT45A1*-*CT45A6*) and their amino-acid sequences share more than 98% identity; hence we cloned one of them (*CT45A1*) to represent all members. Consistently, siRNAs used in this study were designed to target all six *CT45A* family genes. Recently, the *CT45A* family has been updated to 10 genes in NCBI, and their amino-acid sequences still share more than 98% identity [54], making our initial experimental designs still valid. We overexpressed *CT45A* in MDA-MB-231 and MCF7 breast cancer cells and found that CT45A could promote cell migration, EMT signaling and its downstream tumorigenic, EMT, stemness and metastasis related gene expression, indicating that CT45A plays an important role in promoting breast cancer metastasis.

A previous study showed that CT45A has a DEAD/H box with RNA helicase activity and putative nucleic acid binding function [52]. RNA helicases of DEAD box family are required for gene expression and transcription by interacting with RNA polymerase II (Pol II) [64], whether CT45A interacts with RNA Pol II or other transcription factors to promote tumorigenesis and metastasis remains to be further elucidated.

This study investigated the correlation between Uev1A and CT45A in breast cancer cell migartion and EMT signaling. It was found that Uev1A upregulates *CT45A* expression in a Ubc13-dependent manner in one colorectal cancer and two breast cancer cell lines. In a reverse expreriment, depletion of Uev1A in the above three cancer cell lines significantly inhibited the upregulation of *CT45A*, indicating that Uev1A plays a critical role in the upregulation of *CT45A*. Siminarly, Uev1A positively regulates the expression of *CT45A* downstream tumorigenic, EMT, stemness and metastatis related genes in breast cancer cells. Moreover, consistent with their relative transcript levels, we found that N-cadherin was readily detectable in MDA-MB-231 but not MCF7 cells, while E-cadherin was detected in MCF7 but not MDA-MB-231 cells, indicating that these two cell lines regulate EMT by different mechanisms. Furthermore, ectopic expression of *CT45A* could further increase N-cadherin in MDA-MB-231 cells and decrease E-cadherin in MCF7 cells, both of which are expected to promote metastasis. Indeed, *CT45A* depletion in *UEV1A*-overexpressed cells reduced EMT signaling and cell migration to a level comparable to that of control-transfected cells. These findings together indicate that Uev1A is a critical regulator of CT45A-induced cell migration and EMT signaling in breast cancer.

**Fig. 8:**
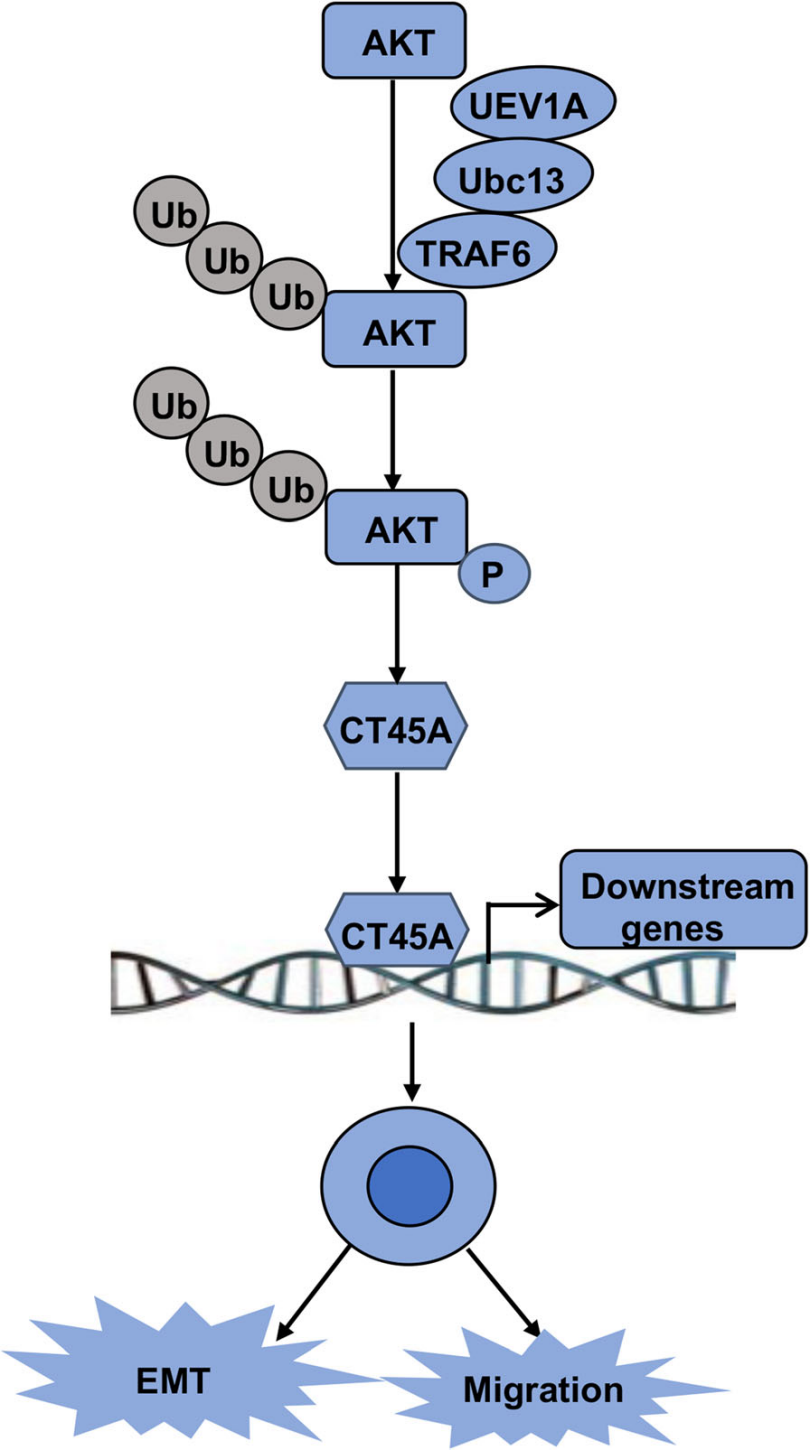
A proposed model in which Uev1A promotes cell migration and EMT through the Uev1A/Ubc13-AKT-CT45A axis in breast cancer. The Uev1A-Ubc13 complex (E2) together with TRAF6 (E3) ubiquitinate AKT, which is essential for the AKT membrane localization, phosphorylation and activation. The phosphorylated and activated AKT positively regulates *CT45A* expression that in turn regulates the expression of *CT45A* downstream genes, leading to increased cell migration and EMT in breast cancer

In order to determine through which signaling pathway(s) Uev1A upregulates *CT45A* expression, we treated *UEV1A* ectopic expression cells with NF-κB and PI3K/AKT pathway inhibitors and found that inhibition of AKT markedly decreased *CT45A* expression, while inhibition of the NF-κB activity had no observable effects. To further confirm that *CT45A* is a direct downstream gene of the AKT pathway, we treated breast cancer cells with the AKT pathway activator IGF-1 and found that the IGF-1 treatment leads to *CT45A* induction. The AKT signaling pathway is closely associated with many biological processes such as cell proliferation, migration and differentiation [24]. It has been reported that AKT undergoes the TRAF6-triggered K63-linked polyubiquitination, which is critical for AKT membrane localization, phosphorylation and subsequent activation [65, 66]. Since Uev1A-Ubc13 is the only known E2 complex to regulate K63-linked polyubiquitination leading to the AKT pathway activation in breast cancer [6], this study reveals a novel Uev1A/Ubc13-AKT-CT45A axis to promote breast cancer cell migration and EMT signaling (Fig. 8). Given limited but consistent observations in a colorectal cancer cell line, the above signaling cascade may be expanded to other types of cancers.

## Conclusion

Overexpression of *UEV1A* is sufficient to activate the AKT pathway in breast cancer cell lines, which in turn upregulates *CT45A* expression to promote breast cancer cell migration and EMT signaling. These observations provide a potential therapeutic target in the treatment of breast cancer.

## Supplementary Information

**Additional file 1: Table S1.** Primers used for quantitative real-time RT-PCR (qRT-PCR). **Table S2.** Upregulated genes in *UEV1A*-overexpressed MDA-MB-231 breast cancer cells (fold change > 5).

**Additional file 2: Fig. S1.** Characterization of *CT45A* family members. **Fig. S2.** Detection of endogenous CT45A. **Fig. S3**. The ectopic expression of *UEV1A* and *UEV1A-F38E*. **Fig. S4.** Efficacy of depleting Uev1A and CT45A in MDA-MB-231 and MCF7 breast cancer cells. **Fig. S5.** Relative *UEV1A* and *CT45A* mRNA levels in HCT116 colorectal cells. **Fig. S6.** Relative transcript levels of *N-cadherin* and *E-cadherin* in MDA-MB-231 and MCF7 cells. **Fig. S7.** Inhibition of the NF-κB pathway by Bay11-7082 treatment. **Fig. S8**. Effects of AKT1 depletion on the *CT45A* expression.

## Data Availability

The datasets used and/or analyzed during the current study are available from the corresponding author upon reasonable request.

## Abbreviations

AKT: Protein kinase B
CTA: Cancer/testis antigen
DMEM: Dubecco’s modified Eagle medium
Dox: Doxycycline
EMT: Epithelial-mesenchymal transition
FBS: Fetal bovine serum
IGF: Insulin-like growth factor
ORF: Open reading frame
qRT-PCR: Real-time reverse-transcription PCR
Ub: Ubiquitin
UEV: Ubiquitin-conjugating enzyme variant.
