# Supplementary material for: Uev1A promotes breast cancer cell migration by up-regulating CT45A expression via the AKT pathway
Source: BMC Cancer. 2021 Sep 9;21:1012. doi: 10.1186/s12885-021-08750-3 (PMC8431945; doi:10.1186/s12885-021-08750-3)
Supplement: Supplementary file 1 — Additional file 1: Table S1. Primers used for quantitative real-time RT-PCR (qRT-PCR). Table S2. Upregulated genes in UEV1A-overexpressed MDA-MB-231 breast cancer cells (fold change > 5). [file 12885_2021_8750_MOESM1_ESM.docx]

**Supplementary file 1: Supplementary tables**

**Table S1** Primers used for quantitative real-time RT-PCR (qRT-PCR)

| Gene name | Primer name | Primers sequences (5’-3’) |
| --- | --- | --- |
| *CT45A* | Forw  Rev | TCGAAATGCTTGAAGGAGTG  TACATCTTGCTGCTTCCTTGA |
| *UEV1A* | Forw  Rev | GAGAGGTTCAAGCGTCTTACCTGAA  ACTGTGCCATCTCCTACTCCTTTCT |
| *TWIST1* | Forw  Rev | CATCCTCACACCTCTGCATT  TTCCTTTCAGTGGCTGATTG |
| *ALDH1A1* | Forw  Rev | GATCCAGGGCCGTACAATAC  CAAATGAGCATAACCAACGG |
| *KIT* | Forw  Rev | ACAAAGAGCAAATCCATCCC  CAATAATGCACATCATGCCA |
| *MAGED4B* | Forw  Rev | GGAGAGGGCAAATAAGTTGG  CGTTGCTCGTTCAATGATCT |
| *HOXB6* | Forw  Rev | CCAGCTACCGCTCTATTCGT  GCGGGTAATAGGAGGAAGTG |
| *HOXD13* | Forw  Rev | ATACGAGCCCTTACCAGCAC  GTGCAGTACACCTGGCTGTT |
| *RASGEF1A* | Forw  Rev | CCATCATCTCTGGCATGAAC  GGCTGTACGGTAGTTGCAGA |
| *SULF2* | Forw  Rev | GACACATCGGTGCTACATCC  AGGGTTTCAATCTCGTGGTC |
| *CXCR4* | Forw  Rev | GCCCTAGCTTTCTTCCACTG  TTGGAGAGGATCTTGAGGCT |
| *N-cadherin* | Forw  Rev | AGCCAACCTTAACTGAGGAGT  GGCAAGTTGATTGGAGGGATG |
| *E-cadherin* | Forw  Rev | CCCATCAGCTGCCCAGAAAATGAA  CTGTCACCTTCAGCCATCCTGTTT |
| *Vimentin* | Forw  Rev | GACAATGCGTCTCTGGCACGTCTT  TCCTCCGCCTCCTGCAGGTTCTT |
| *AKT1* | Forw  Rev | TCCTCCTCAAGAATGATGGCA  GTGCGTTCGATGACAGTGGT |
| *AKT2* | Forw  Rev | GCAAAGAGGGCATCAGTGAC  CTCGTGGTCCTGGTTGTAGA |
| *AKT3* | Forw  Rev | GGTGCAGAGTCCCCTAGAGA  AGATTTTGGCGACAGCAGGA |
| *GADPH* | Forw  Rev | GAAGGTGAAGGTCGGAGTC  GAAGATGG TGATGGGATTTC |

**Table S2** Upregulated genes in *UEV1A*-overexpressed MDA-MB-231 breast cancer cells (fold change > 5)

| Gene ID | Fold Change | Gene Description |
| --- | --- | --- |
| 541465 | 224.6806 | cancer/testis antigen CT45-6 |
| 441521 | 131.8982 | cancer/testis antigen CT45-5 |
| 441519 | 107.5529 | cancer/testis antigen CT45-3 |
| 7032 | 86.1545 | trefoil factor 2 (spasmolytic protein 1) (TFF2) |
| 114798 | 47.0413 | SLIT and NTRK-like family, member 1 (SLITRK1) |
| 728911 | 40.7197 | cancer/testis antigen CT45-2 |
| 8722 | 35.0693 | cathepsin F (CTSF) |
| 90737 | 30.2083 | P antigen family, member 5 (prostate associated) (PAGE5) |
| 6035 | 29.5467 | ribonuclease, RNase A family, 1 (pancreatic) (RNASE1) |
| 9840 | 29.4148 | thymocyte expressed, positive selection associated 1 (TESPA1) |
| 56143 | 29.0436 | protocadherin alpha 5 (PCDHA5) |
| 2577 | 28.2247 | G antigen 5 (GAGE5) |
| 3887 | 27.9001 | keratin, hair, basic, 1 (KRTHB1) |
| 53826 | 26.7176 | FXYD domain containing ion transport regulator 6 (FXYD6) |
| 2576 | 26.147 | G antigen 4 (GAGE4) |
| 8477 | 24.2815 | G protein-coupled receptor 65 (GPR65) |
| 4102 | 23.1769 | melanoma antigen family A, 3 (MAGEA3) |
| 4105 | 22.627 | melanoma antigen family A, 6 (MAGEA6) |
| 2574 | 21.1109 | G antigen 2 (GAGE2) |
| 26749 | 20.8572 | G antigen 2E (GAGE2E) |
| 2578 | 18.1914 | G antigen 6 (GAGE6) |
| 6347 | 16.6346 | chemokine (C-C motif) ligand 2 (CCL2) |
| 2543 | 16.5282 | G antigen 1 (GAGE1) |
| 4642 | 15.9014 | myosin ID (MYO1D) |
| 3889 | 15.615 | keratin, hair, basic, 3 (KRTHB3) |
| 196051 | 13.2882 | phosphatidic acid phosphatase type 2 domain containing 1A (PPAPDC1A) |
| 3641 | 12.7966 | insulin-like 4 (placenta) (INSL4) |
| 57447 | 11.5288 | NDRG family member 2 (NDRG2) |
| 2556 | 11.3839 | gamma-aminobutyric acid (GABA) A receptor, alpha 3 (GABRA3) |

| 3294 | 9.7785 | hydroxysteroid (17-beta) dehydrogenase 2  (HSD17B2) |
| --- | --- | --- |
| 623 | 9.6186 | bradykinin receptor B1 (BDKRB1) |
| 945 | 9.1698 | CD33 molecule (CD33) |
| 2331 | 8.8503 | fibromodulin (FMOD) |
| 9535 | 8.3232 | glia maturation factor, gamma (GMFG) |
| 84707 | 8.1616 | brain expressed X-linked 2 (BEX2) |
| 7433 | 8.1194 | vasoactive intestinal peptide receptor 1 (VIRP1) |
| 57407 | 7.8678 | HSCARG protein (HSCARG) |
| 57451 | 7.4394 | odz, odd Oz/ten-m homolog 2 (Drosophila) (ODZ2) |
| 3119 | 7.2527 | major histocompatibility complex, class II, DQ beta 1 (HLA-DQB1) |
| 7078 | 7.0654 | TIMP metallopeptidase inhibitor 3 (Sorsby fundus dystrophy, pseudoinflammatory) (TIMP3) |
| 7745 | 6.9846 | zinc finger protein 192 (ZNF192) |
| 389860 | 6.8923 | P antigen family, member 2B (PAGE2B) |
| 203569 | 6.2991 | P antigen family member 2 (PAGE2) |
| 794 | 6.1328 | calbindin 2, 29kDa (calretinin) (CALB2) |
| 55799 | 5.7369 | calcium channel, voltage-dependent, alpha 2/delta 3 subunit (CACNA2D3) |
| 6695 | 5.4644 | sparc/osteonectin, cwcv and kazal-like domains proteoglycan (testican) 1 (SPOCK1) |
| 8862 | 5.2982 | apelin, AGTRL1 ligand |
|  |  |  |
