# Supplementary material for: Uev1A promotes breast cancer cell migration by up-regulating CT45A expression via the AKT pathway
Source: BMC Cancer. 2021 Sep 9;21:1012. doi: 10.1186/s12885-021-08750-3 (PMC8431945; doi:10.1186/s12885-021-08750-3)
Supplement: Supplementary file 2 — Additional file 2: Fig. S1. Characterization of CT45A family members. Fig. S2. Detection of endogenous CT45A. Fig. S3. The ectopic expression of UEV1A and UEV1A-F38E. Fig. S4. Efficacy of depleting Uev1A and CT45A in MDA-MB-231 and MCF7 breast cancer cells. Fig. S5. Relative UEV1A and CT45A mRNA levels in HCT116 colorectal cells. Fig. S6. Relative transcript levels of N-cadherin and E-cadherin in MDA-MB-231 and MCF7 cells. Fig. S7. Inhibition of the NF-κB pathway by Bay11-7082 treatment. Fig. S8. Effects of AKT1 depletion on the CT45A expression. [file 12885_2021_8750_MOESM2_ESM.docx]

**Supplementary file 2: Supplementary figures**

**Supplementary figure legends**

**Fig. S1.** Characterization of *CT45A* family members. (**a**) Altered expression of genes encoding cancer/testis antigens in *UEV1A*-overexpressed MDA-MB-231 cells. Results were extracted from microarray data and the red line indicates 25-fold induction threshold. (**b**) The amino acid sequence alignment of 10 *CT45A* family genes.

**Fig. S2.** Detection of endogenous CT45A. Western blot analysis of endogenous CT45A proteins in different cell lines including MDA-MB-231, MCF7, HCT116, 293T, HeLa and U2OS by using a commercial anti-CT45A polyclonal antibody. Red asterisks indicate possible CT45A protein bands based on estimated molecular weights..

**Fig. S3**. The ectopic expression of *UEV1A* and *UEV1A-F38E*. The ectopic expression of *UEV1A* and *UEV1A-F38E* in **(a)** MDA-MB-231 and (**b**) MCF7 cells were detected by western blot using an HA-tag antibody. CK, control treatment. The gel images are cropped from available original blots.

**Fig. S4.** Efficacy of depleting Uev1A and CT45A in MDA-MB-231 and MCF7 breast cancer cells. (**a,b**) MDA-MB-231 (**a**) and MCF7 (**b**) cells were transfected with shRNA against Uev1A (shUEV1A) and non-specific target (shCK). The transcript levels of *UEV1A* were determined by qRT-PCR. (**c,d**) MDA-MB-231 (**c**) and MCF7 (**d**) cells were transfected with siRNA against CT45A (siCT45A) and non-specific target (siCK). The transcript levels of *CT45A* were determined by qRT-PCR. shCK, control shRNA; siCK, control siRNA. All experiments were performed in at least triplicate, and the results are the average with standard deviation showing as error bars. **, *P*<0.01.

**Fig. S5.** Relative *UEV1A* and *CT45A* mRNA levels in HCT116 colorectal cancer cells. (**a**) HCT116 cells were transfected with shRNA against Uev1A (shUEV1A) and non-specific target (shCK). The *UEV1A* transcript levels were determined by qRT-PCR. (**b,c**) The expression levels of *CT45A* (**b**) and *UEV1A* (**c**) in HCT116 cells overexpressing *UEV1A* and depleting CT45A were monitored by qRT-PCR. shCK, control shRNA; siCK, control siRNA. All experiments were performed in at least triplicate, and the results are the average with standard deviation showing as error bars. **, *P*<0.01.

**Fig. S6.** Relative transcript levels of *N-cadherin* and *E-cadherin* in MDA-MB-231 and MCF7 cells. (**a**) Relative *N-cadherin* transcript levels in MDA-MB-231 and MCF7 cells. (**b**) Relative *E-cadherin* transcript levels in MDA-MB-231 and MCF7 cells. Data came from the same source as shown in Fig. 6a,b, in which *N-cadherin* and *E-cadherin* transcripts levels in both cell lines were relative to their own endogenous *GAPDH* transcript level. ***, *P*<0.001.

**Fig. S7.** Inhibition of the NF-κB pathway by Bay11-7082 treatment. MDA-MB-231 (**a**), MCF7 (**b**) and HCT116 (**c**) cells transiently transfected with *UEV1A* were treated with or without 30 μM Bay11-7082 for 24 hrs. The nuclear fraction (N) was then prepared for western blot analysis using an anti-p65 antibody to assess the nuclear translocation of p65, while the whole cell extract (WCE) was prepared to monitor Uev1A levels by western blot against an HA-tag antibody. The gel images in are cropped from available original blots and numbers underneath the WB images indicate relative band intensity after normalization with the loading control. The *CT45A* mRNA levels in MDA-MB-231 (**d**), MCF7 (**e**) and HCT116 (**f**) cells transiently transfected with *UEV1A* were treated with or without 30 μM Bay11-7082 followed by qRT-PCR. CK, control treatment; Bay, Bay11-7082. All experiments were performed in at least triplicate, and the results are the average with standard deviation showing as error bars. **, *P*<0.01.

**Fig. S8.**  Effects of AKT1 depletion on the *CT45A* expression. (**a,b**) Relative transcript levels of endogenous *AKT1*, *AKT2* and *AKT3* in MDA-MB-231 (**a**) and MCF7 (**b**) cells as measured by qRT-PCR, in comparison to their own *GADPH* expression. (**c,d**) MDA-MB-231(**c**) and MCF7 (**e**) cells were transfected with siRNA against AKT1 and non-specific siRNA (siNC). Cellular AKTs were detected by western blot against an anti-AKT antibody. The gel images in are cropped from available original blots and numbers underneath the WB images indicate relative band intensity after normalization with the loading control. The asterisk indicates non-specific bands whose intensity was not altered by siAKT1 treatment. (**e,f**) MDA-MB-231 (**e**) and MCF7 (**f**) cells were transfected with siRNA against AKT1 and non-specific siRNA (siNC). followed by qRT-PCR to measure *CT45A* mRNA levels. siNC, control siRNA. All experiments were performed in at least triplicate, and the results are the average with standard deviation showing as error bars. *, *P*<0.05. **, *P*<0.01. ***, *P*<0.001.


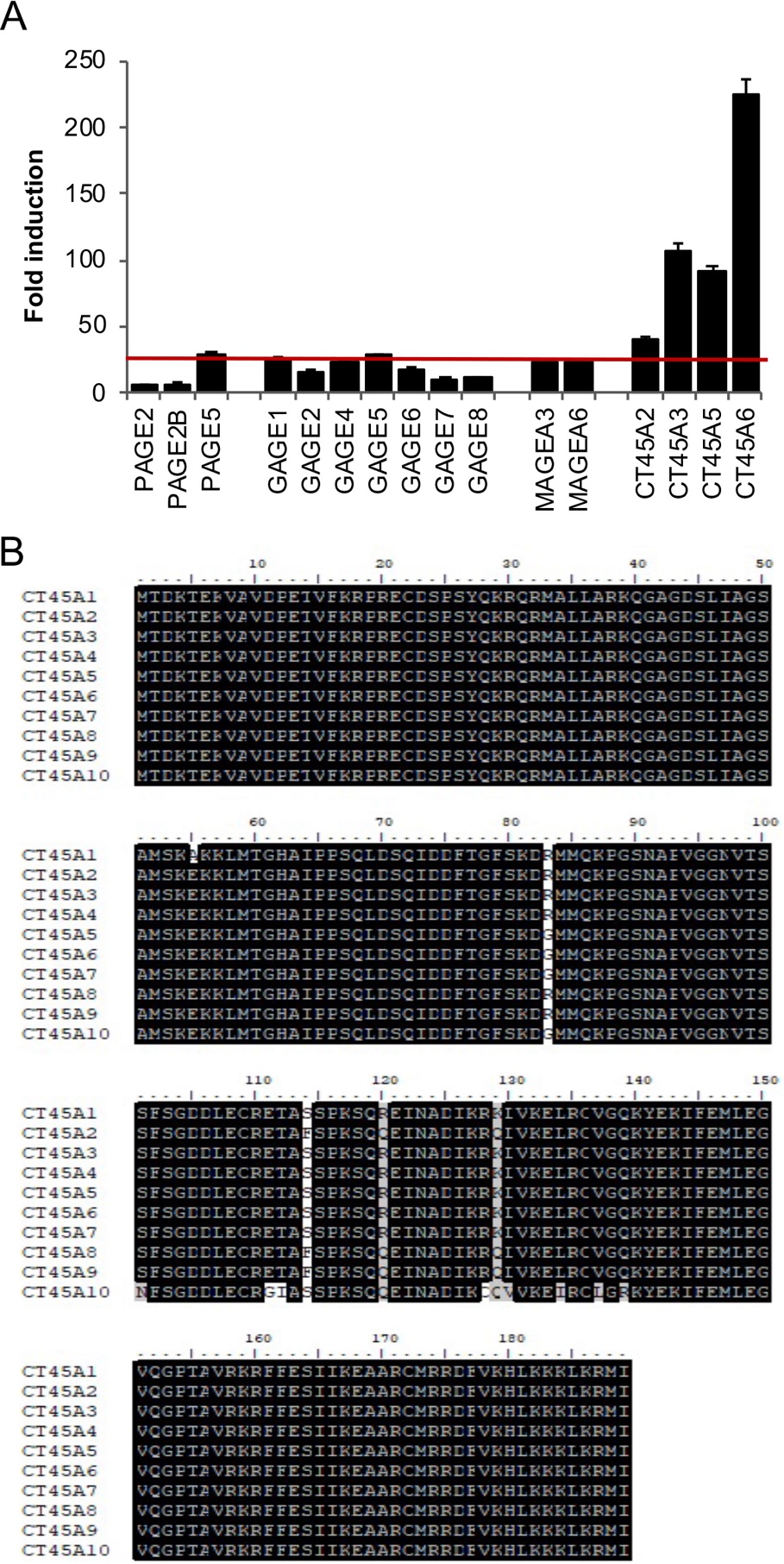


Figure S1

Figure S2


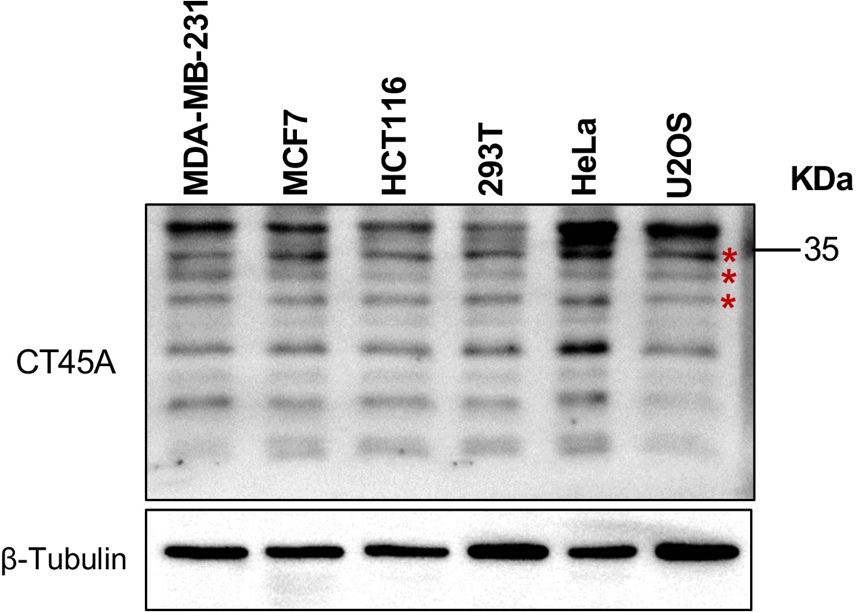


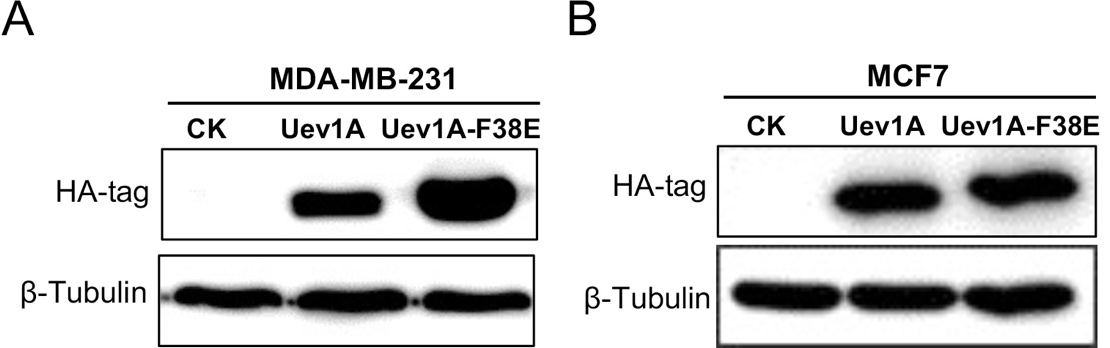


Figure S3


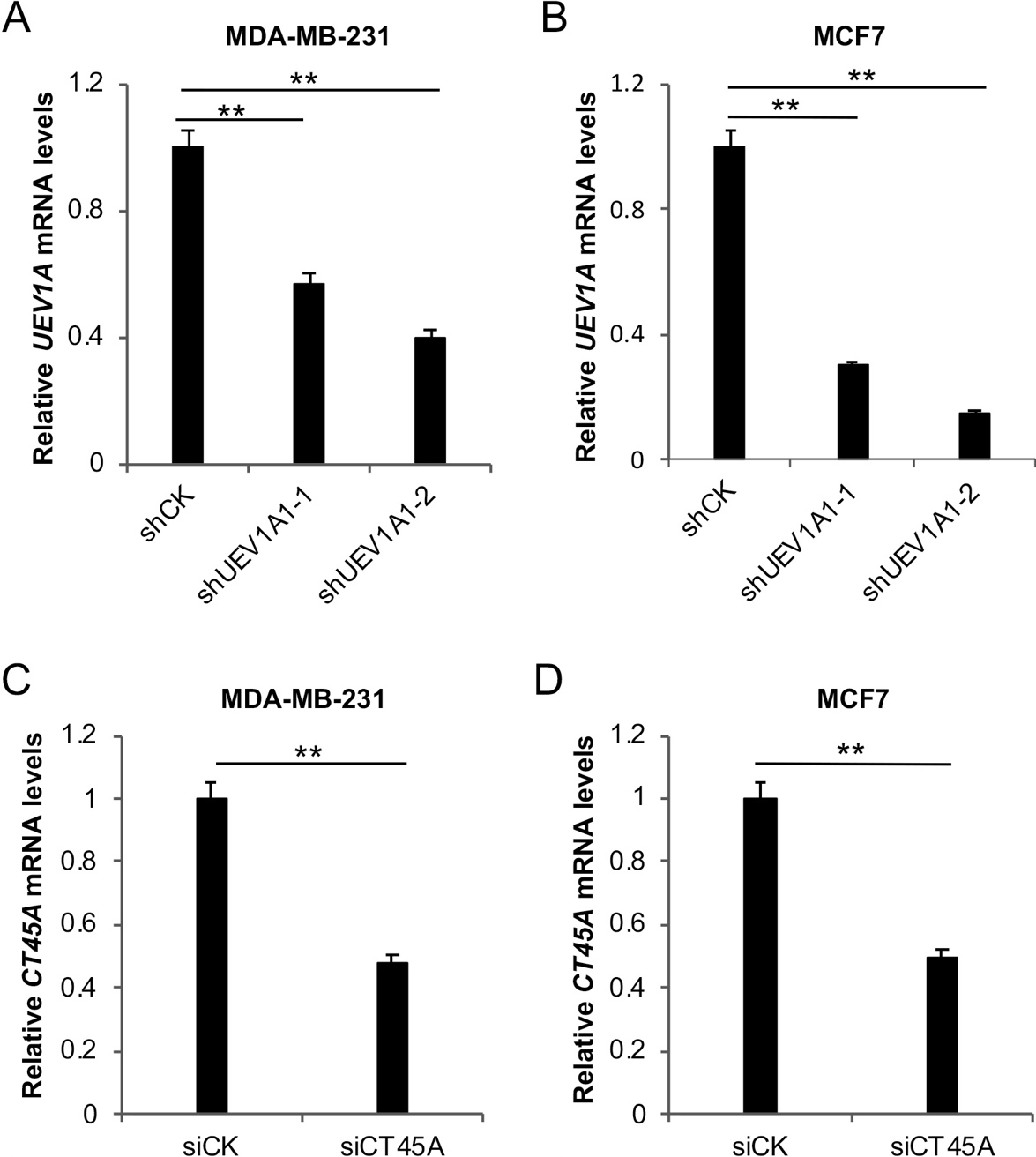


Figure S4


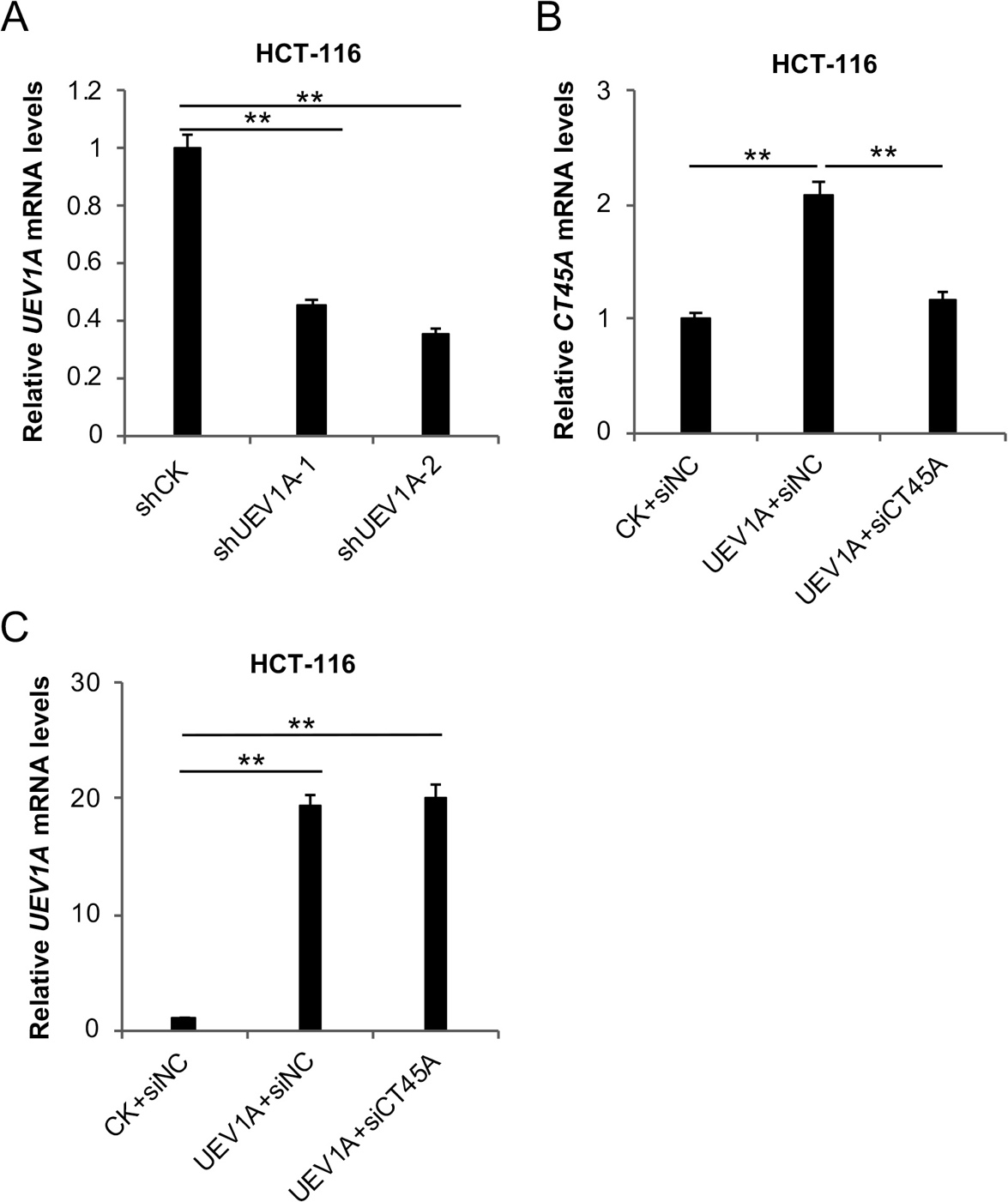


Figure S5


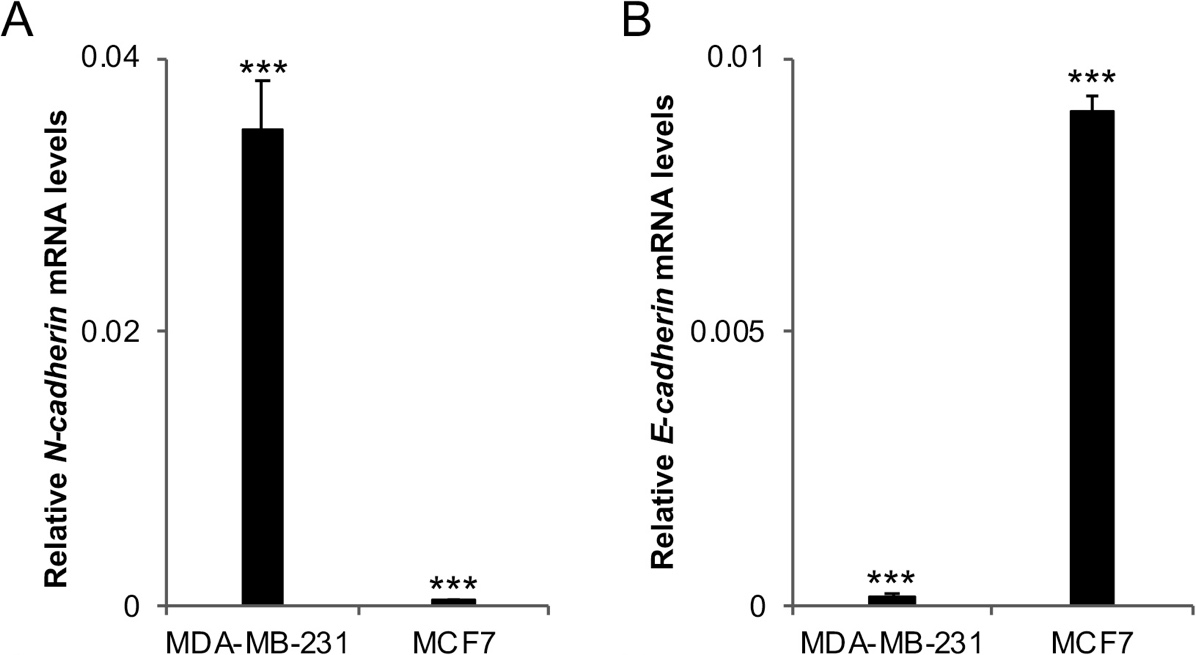


Figure S6


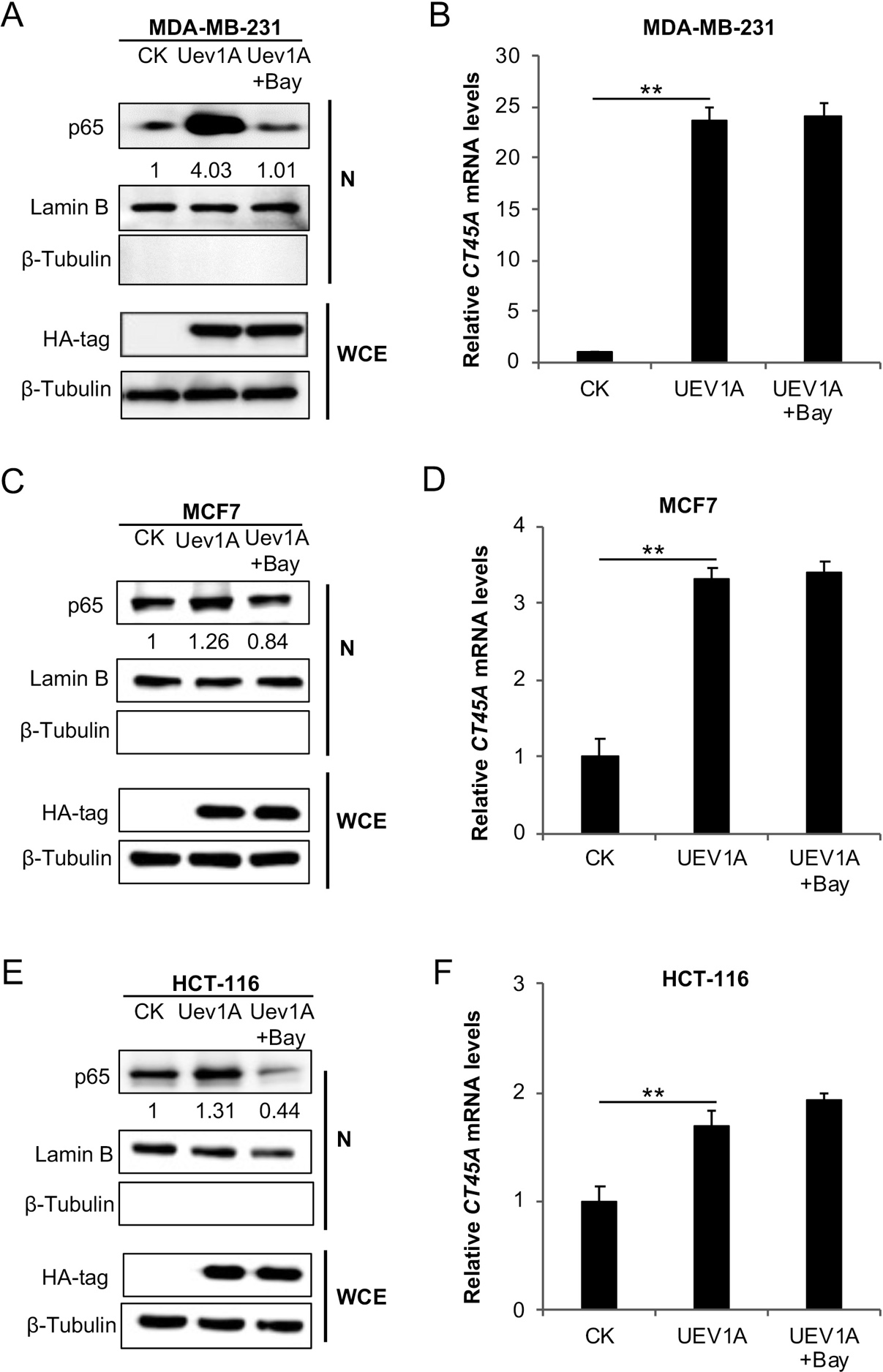


Figure S7


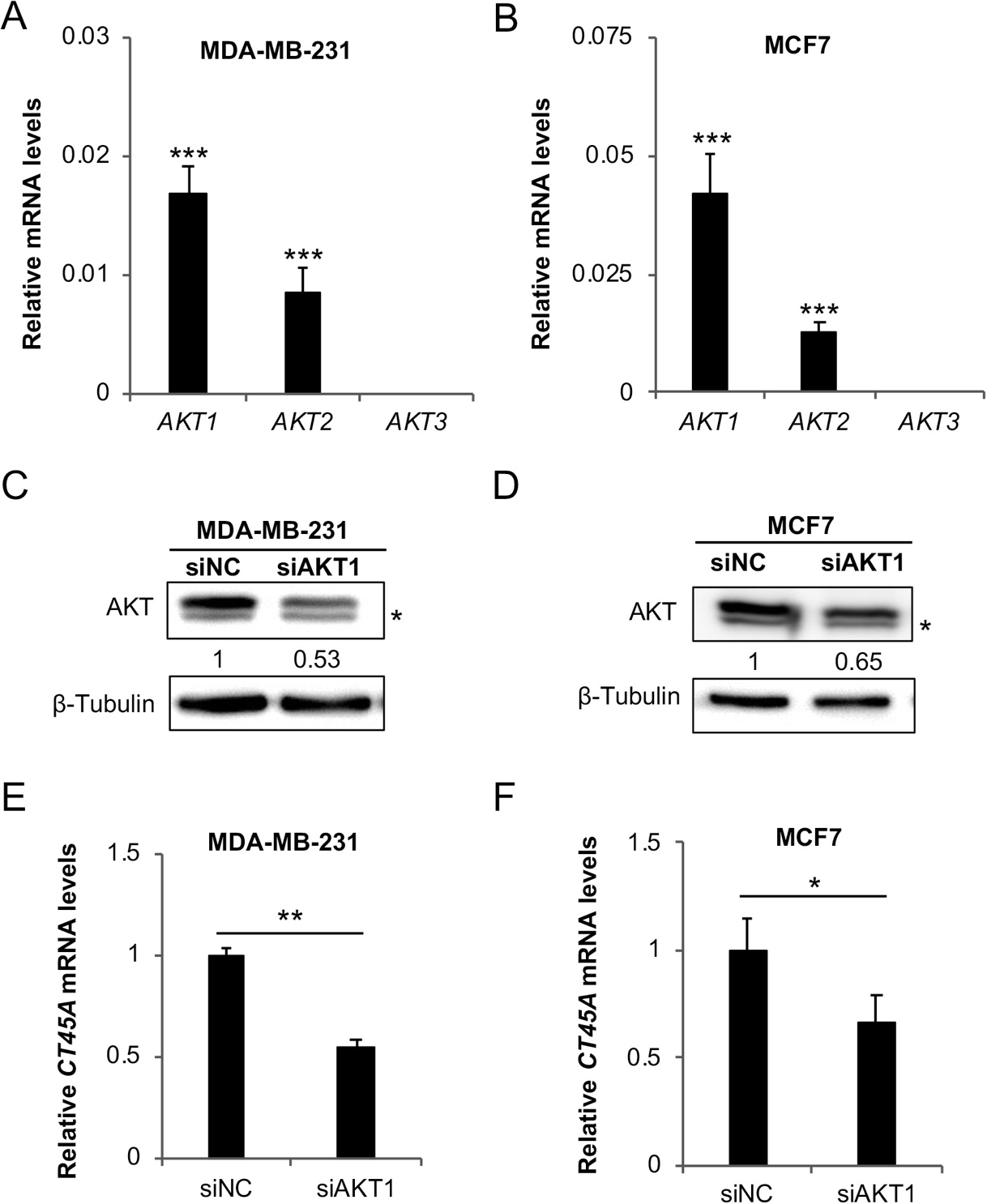


Figure S5

Figure S8
